# Testing two digital stress-management interventions in a randomized controlled trial of breast cancer patients

**DOI:** 10.1038/s41598-025-22889-0

**Published:** 2025-11-06

**Authors:** Karianne Svendsen, Lise Solberg Nes, Sigrid Leithe, Anders Meland, Ylva M. Gjelsvik, Elin Børøsund, Ine M. Larsson, Tor Åge Myklebust, Aina Balto, Christine M. Rygg, Cecilie E. Kiserud, Michael H. Antoni, Trudie Chalder, Ingvil Mjaaland, Linda E. Carlson, Hege R. Eriksen, Giske Ursin

**Affiliations:** 1 Cancer Registry of Norway, Norwegian Institute of Public Health, Oslo, Norway; 2 Lipid Clinic, Oslo University Hospital, Oslo, Norway; 3 Department of Digital Health Research, Division of Medicine, University Hospital, Oslo, Norway; 4 Institute of Clinical Medicine, Faculty of Medicine, University of Oslo, Oslo, Norway; 5 Department of Psychiatry and Psychology, College of Medicine and Science, Rochester, USA; 6 Department of Sport and Social Sciences, Norwegian School of Sport Sciences, Oslo, Norway; 7 Department of Nursing and Health Sciences, Faculty of Health and Social Sciences, University of South-Eastern Norway, Drammen, Norway; 8 Department of Oncology, Oslo University hospital, Oslo, Norway; 9 Department of Psychology, Cancer Control Program, Sylvester Comprehensive Cancer Center, University of Miami, Miami, FL US; 10 Department of Psychological Medicine, King’s College London, London, UK; 11 Department of Oncology and Hematology, Stavanger University Hospital, Stavanger, Norway; 12 Departments of Oncology and Psychology, University of Calgary, Calgary, Canada; 13 Department of Sport, Food and Natural Sciences, Western Norway University of Applied Sciences, Bergen, Norway; 14 Department of Preventive Medicine, Keck School of Medicine, University of Southern California, Los Angeles, CA USA; 15 Department of Nutrition, University of Oslo, Oslo, Norway

**Keywords:** Breast cancer, psychosocial, Stress-management, Mindfulness, Cognitive behavioral, Late effects., Cancer, Breast cancer, Cancer therapy, Health services, Quality of life

## Abstract

The digital stress-management intervention StressProffen has been shown to be associated with improved well-being and quality of life for cancer survivors. In the Coping After Breast Cancer (CABC) trial, effects of 6 months’ access to modified versions of StressProffen, delivered through a digital download-only model, were examined. Women with breast cancer were invited to participate in the trial 6–9 months following diagnosis. Eligible participants were randomized to either: (1) digital cognitive behavioral therapy stress-management intervention (CBI), *n* = 140, (2) digital mindfulness-based stress-management intervention (MBI), *n* = 143, or (3) usual-care (control group), *n* = 147. Primary outcome was change in perceived stress level (PSS-10), while secondary outcomes included changes in health-related quality of life (HRQoL), anxiety and depression, fatigue, mindfulness, sleep and coping. Perceived stress level at baseline was low for all groups. No statistically significant mean differences (MD) were detected between either of the intervention groups and the control group from baseline to 6-month follow-up for perceived stress level (MBI: MD -0.28 [95%CI: -1.75, 1.19], CBI: MD -0.42 [95%CI: -1.89, 1.06]), nor for the majority of the secondary outcomes. After 6 months of access, the CBI and MBI stress-management interventions did not yield significantly improved outcomes for women with breast cancer compared with usual-care controls. Further explorations of which interventions and delivery models may optimize use and effect, best timing for delivery, and individual preferences are needed. ClinicalTrials.gov identifier NCT04480203.

**Supplementary Information:**

The online version contains supplementary material available at 10.1038/s41598-025-22889-0.

## Background

Progress in diagnostics and treatment have raised the 5-year relative survival rate for early detected breast cancer to above 90% in many Western countries^1,2^. Although improvements in supportive therapies have alleviated the immediate side-effects of adjuvant systemic therapies, receiving a breast cancer diagnosis and undergoing subsequent treatment is associated with high levels of stress, including, but not limited to, uncertainty about treatment and treatment outcome, physical symptoms such as pain, nausea and fatigue, and fear of death, disfiguration and recurrence^3^. These types of stressors can persist long after the person is considered cancer-free^3,4^, and can induce psychosocial and physical challenges that may impact overall psychological well-being, health-related quality of life (HRQoL) and ability to cope (i.e., manage or adjust to the stressor exposure)^3–11^. Coping is defined as positive response outcome expectancy in accordance with the Cognitive Activation Theory of Stress^12^.

Psychosocial stress-management interventions have been shown to be effective in women with breast cancer for decades, being associated with reduced stress, anxiety and depression, fatigue, insomnia, pain and enhanced HRQoL^3,13–18^. These interventions often incorporate cognitive-behavioral and mindfulness techniques^6,8^, that are, separately and combined, known to be effective in reducing stress and cancer-related side effects, as well as having positive effects on HRQoL^3,17,19–24^. While the aim of cognitive-behavioral therapy (CBT) is to modify cognitions and behavior with brief- and goal oriented interventions in order to facilitate adjustment and coping^3^, one aim of mindfulness-based interventions is to learn to tolerate and change ones relationship to stressful thoughts, feelings or sensations, which over time can lead to reduced physiological over-arousal^25^. Some reviews exist comparing the effect of various intervention types in support of stress-management in cancer development^26–29^, but few studies have sought to directly compare CBT and mindfulness effects in randomized controlled trials (RCTs)^8,30^.

As the outreach of evidence-based psychosocial interventions is often limited (e.g., due to geographical limitations), the use of electronic health (eHealth) solutions to deliver psychosocial stress-management interventions to breast cancer patients has been increasing^31,32^. In a two-arm 12-month RCT, StressProffen^33,34^, an evidence-based, user-centered application (app)-based stress-management program, delivered in a simple blended care model, has shown to be associated with reduced stress and symptoms of anxiety and depression and improved capacity for self-regulation and HRQoL in cancer survivors (majority breast cancer)^33–36^. Most psychosocial stress-management interventions shown to be effective were initially CBT-based^3^, and StressProffen is subsequently primarily CBT-based, with elements of mindfulness^33–36^. As studies comparing recognized psychosocial treatment methods are lacking^8^, the current research team in the Coping After Breast Cancer (CABC) project developed two distinct adaptations of the original StressProffen app, with either CBT content; the cognitive behavioral intervention (CBI) or mindfulness content; the mindfulness-based intervention (MBI), seeking to test both against usual care in a new delivery format (i.e., entirely self-administered)^37^. Participants receiving either StressProffen CBI or MBI over 6 months, compared to participants receiving usual-care (i.e., control group) were hypothesized to experience decreased perceived stress level, anxiety and depression and fatigue, and improvements in HRQoL, mindfulness, coping and sleep.

## Methods

### Study design and participants

The protocol and study design of the digital CABC trial has been fully described previously^38^. The trial was first submitted to ClinicalTrials.gov 09/07/2020 with identifier NCT04480203.

Women diagnosed with either ductal carcinoma in situ, or breast cancer stage I-III, human epidermal growth factor receptor 2–positive (HER2-positive) or estrogen receptor–negative tumor(s)) (ER-negative) registered in the Cancer Registry of Norway (CRN), aged 21–69 years who had completed the digital CRN patient reported outcome measures (CRN PROMs) survey (sent to all breast cancer patients at least 21 days following diagnosis^39^ were eligible for trial inclusion. Recruitment was restricted to women with these cancer subtypes in order to avoid overlap with ongoing clinical trials of breast cancer treatment^38^.

In total 1123 potentially eligible breast cancer patients were identified from the CRN PROMs survey and received the CABC trial specific survey 6–9 months following their diagnosis and in the period January 2021 - May 2023. In total 467 completed the CABC survey and provided consent for participating in a clinical trial testing one of two different stress-management interventions. Participants were informed about the structure of the interventions, including the number and duration of modules. They did, however, not receive information about the content of the two interventions.

Women who consented were randomized 1:1:1 to CBI, MBI or usual-care controls. The randomization was concealed, and we employed a block randomization variant without stratification, with block sizes varying between 12 and 27. The output of the randomization algorithm was either an access code for downloading the intervention app, or a code indicating that the participant was randomized to the control group. The randomization was performed using R studio version 1.2.1335 by a project assistant who had no influence on the intervention allocation. Twenty-three participants were included in a pilot study conducted prior to the start of the trial^38^ and were thus excluded from the study sample.

**Fig. 1:**
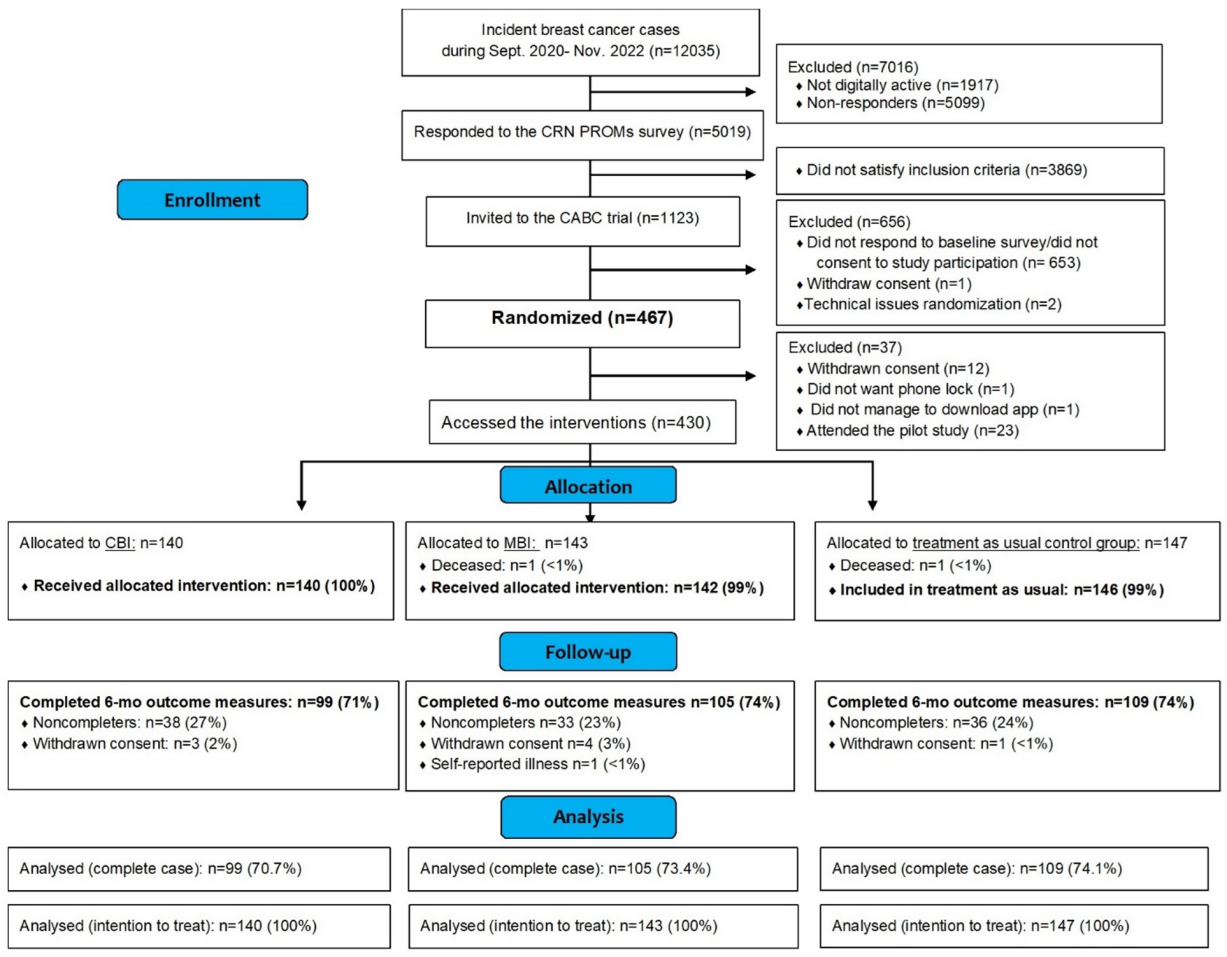
Participant trial flow chart. *CRN PROMs* Cancer Registry of Norway (CRN) patient reported outcome measures (survey sent to all digitally active breast cancer patients after diagnosis), *CABC* Coping After Breast Cancer, *CBI* cognitive behavioral therapy intervention, *MBI* mindfulness-based intervention.

In an initial, introductory call to participants in the intervention groups, three additional eligibility criteria were assessed: Whether the participants; (1) understood Norwegian, (2) had access to a smartphone or tablet, and (3) had/were willing to enable phone locks on their devices (a necessity for accessing the app). In total 430 satisfied the eligibility criteria and participants randomized to CBI (*n* = 140) or MBI (*n* = 143) accessed the interventions (downloaded the StressProffen app with intervention specific content on their phones with guidance from study staff), whereas participants randomized to controls (*n* = 147) received no more follow-up (Fig. 1).

### Intervention arms and delivery model

The CBI and MBI interventions,  commencing from the original StressProffen program (i.e., primarily CBT based but with aspects of mindfulness), were developed to be almost identical in length, design, format and layout, for details see^37^. Each intervention consisted of 10 modules with 9–16 brief steps containing various stress-management related educational material and exercises (i.e., diaphragmatic breathing, visualization, focus) available through text, audio, video, and illustrations. The interventions are self-guided with a 3-day mandatory pause between modules to encourage reflection and practice. The titles of the 10 modules in both interventions are displayed in Supplementary file 1. Examples of CBI and MBI content are displayed in Supplementary file 2 and 3, respectively.

The original StressProffen program was delivered in a simple blended care model (i.e., one in-person introduction session providing rationale for the stress-management concept and guidance in downloading the app, followed by two follow-up phone calls)^34,35^. Most “health apps” are however downloaded individually from online stores, and are rarely delivered with additional support^40^. The current CABC trial therefore opted to deliver the StressProffen CBI and MBI versions in a digital download-only delivery model. To add a follow-up aspect for the intervention (i.e., to encourage use and adherence), participants were contacted by phone three times during the intervention period (about 1-, 3- and 6-weeks post app access). These follow-up phone calls, although brief, were conducted to ask about status, help resolve any potential technical issues or barriers for use, and through such help motivate continued usage of the app.

### Data collection and outcome measures

All outcomes were assessed with the CABC 1 survey at baseline (6–9 months after diagnosis) and with the CABC 2 survey 6 months after baseline (about 13 months after diagnosis). The exception in CABC 2 was the outcome fatigue that was already scheduled to be assessed with the ongoing CRN PROMs 2 survey 14 months after diagnosis^38^ (Supplementary file 4).

### Outcome measures

#### Primary outcome

The primary outcome, perceived stress, was assessed using Cohen’s 10-item Perceived Stress Scale^41^. Items were rated on a 5-point Likert scale ranging from “never” (0) to “very often (5)” over the last month, and a total score was calculated (0–40). Score 0–13 represents low perceived stress level, 14–26 moderate, and 27–40 high perceived stress level^41^.

#### Secondary outcomes

An *anxiety and depression* score was generated from four individual items in the brief version of the Patient Health Questionnaire for Depression and Anxiety (PHQ4)^42^. *Fatigue* was measured using Chalder’s Fatigue Scale (13 items) and presented as a summary score^43^. *HRQoL* was measured by the RAND Corporation 36-Item Short Form Health Survey (RAND-36). RAND-36 assesses 8 health domains: physical functioning, role limitations caused by physical health problems, role limitations caused by emotional problems, social functioning, emotional well-being, energy/fatigue, pain, and general health perceptions^44^. *Mindfulness* was assessed using the 15 items from Baer’s 5 Facet Mindfulness Questionnaire (FFMQ-15) and presented as a total score^45,46^. *Coping*, helplessness and hopelessness were measured by the Theoretically Originated Measure of the Cognitive Activation Theory of Stress^47^. *Sleep* (duration and quality) was measured by items from the Norwegian Shift Work, Sleep and Health survey^48^. Rationale and more details on outcome measures are provided in the protocol paper^38^.

#### Sociodemographic and medical data

Sociodemographic data (i.e., age, educational level, relationship and family status, income, physical activity level, smoking and alcohol habits, height, and weight) were obtained from the CRN PROMs 1 survey (administered ≥ 21 days following diagnosis). Medical data on breast cancer diagnosis were obtained from the CRN.

#### App use

Data related to program use (i.e., such as app progress/activity, time spent using the app, days from first to last use, number of completed modules, etc.) were automatically extracted from user logs that were collected and stored on a secure research server at the Services for Sensitive Data, University of Oslo.

### Statistical analysis

All analysis followed a statistical analysis plan that was evaluated by the CABC main project group prior to analysis.

The study was powered to find significant differences in the primary outcome (i.e., 6-month between-group difference in perceived stress) using independent sample *t*-tests. Presumed effect size and variance were based on previous results from the StressProffen study^34,49^. Originally, we used an alpha level of 5%, but since there were two intervention groups, we adjusted for multiple comparisons (two intervention groups) and reduced the alpha level to 2.5% in February 2022. Simultaneously, we downgraded one of the two primary outcomes (HRQoL) to a secondary outcome. Assuming a completion rate of 70%, we needed a minimum of 335 participants^38^, and ended up including 430 participants.

Results for all outcomes are presented as the mean change from baseline to 6-month follow-up, adjusted for baseline values. Between-group differences were calculated as the intervention group changes from baseline minus control group change from baseline. Analyses were performed according to the intention-to-treat principle, with all participants included regardless of app use and loss to follow-up. Missing data on outcomes at baseline and follow-up were imputed using fully conditional specification. In addition, results from the complete case analysis are presented in Supplementary file 5. Groups were also compared after controlling for the a-priori defined potential confounders of age (grouped as 18–44, 45–54, 55–69), educational level (primary school, secondary school, college/university ≤ 4 or > 4 years), having children < 18 years (yes/no), BMI (< 25, 25–29 and ≥ 30 kg/m^2^), breast cancer stage (DCIS, stage I, stage II and III, missing) and drinking alcohol (yes/no) in a multi-adjusted regression model. Results from these analyses are presented in Supplementary file 6.

For the primary outcome, exploratory subgroup analyses were performed by app completion (completed 0–6 modules vs. 7–10 modules)^35^, perceived stress at baseline [above vs. below median and low vs. moderate/high levels (due to few individuals in the high group)], age (below vs. above median age of 53 years), and stage (DCIS vs. invasive). P-values are reported for the interactions between subgroup and intervention group to assess whether intervention effects varied by subgroup.

### Multiple imputations

Missing data (including due to deaths [*n* = 2]) on all primary and secondary outcomes at baseline and at follow-up were addressed using multiple imputations by chained equations under the missing-at-random assumption. Each outcome was imputed separately, stratified by randomized group, with all the variables in the multivariable regression model included in the conditional models. Additionally, the EORTC QLQ-C30 emotional functioning scale^50^ from CRN PROMs survey 1 and 2 were included as auxiliary variables due to strong association with non-response on CABC survey 2. This was done to satisfy the missing-at-random assumption and reduce the variance of the imputations^51^. To reduce the complexity of the imputation models, the cross-lagged predictors from the surveys were not included in the conditional distributions^52^.

For the primary outcome, the imputation models were validated by comparing observed and imputed data, and performing posterior predictive checking of the estimated intervention effect^53^. Since the pattern of missingness was similar for all outcomes, this was not repeated for the secondary outcomes. A total of 100 imputed datasets were created, and the estimates of the intervention effect were combined using Rubin’s rules.

## Results

### Sample description

The sociodemographic characteristics were similar between participants in all three randomized groups. Mean age was 53.1 (SD 9.5) in CBI, 51.5 (SD 9.5) in MBI, and 52.6 (SD 9.2) in controls, respectively. There was a higher proportion of individuals in the MBI group who were living with children < 18 years (39.2%) compared to in the control group (29.3%), and a lower proportion of women in the CBI group than in the control group who reported drinking (any) alcohol (57.9% vs. 69.4%) (Table 1).

**Table 1:** Sociodemographic background data for participants in all three groups.

Interventions					
Variables	CBI (*n* = 140)	MBI (*n* = 143)	Controls (*n* = 147)	*P* (CBI vs. Controls)	*P* (MBI vs. Controls)
**Age (years)**				0.59	0.32
Mean (SD)	53.1 (9.5)	51.5 (9.5)	52.6 (9.2)		
Median (min-max)	53.0 (30.0–69.0)	52.0 (26.0–68.0)	53.0 (31.0–70.0)		
**BMI (kg/m** ^**2**^ **)**				0.05	0.72
Mean (SD)	26.4 (5.2)	25.4 (4.2)	25.2 (4.5)		
Median (min-max)	26.0 (16.0–41.0)	25.0 (17.0–35.0)	24.0 (16.0–39.0)		
**Relationship status**,** n (%)**				0.19	0.34
In a relationship	117 (83.6)	115 (80.4)	113 (76.9)		
Not in a relationship	19 (13.6)	21 (14.7)	28 (19.0)		
Missing	4 (2.9)	7 (4.9)	6 (4.1)		
**Children < 18**^a^, **n (%)**				0.40	0.06
No	88 (62.9)	80 (55.9)	98 (66.7)		
Yes	48 (34.3)	56 (39.2)	43 (29.3)		
Missing	4 (2.9)	7 (4.9)	6 (4.1)		
**Educational level**,** n (%)**				0.85	0.42
Primary school	6 (4.3)	5 (3.5)	4 (2.7)		
Secondary school	43 (30.7)	37 (25.9)	41 (27.9)		
College/university ≤ 4 years	48 (34.3)	41 (28.7)	53 (36.1)		
College/university > 4 years	39 (27.9)	53 (37.1)	43 (29.3)		
Missing	4 (2.9)	7 (4.9)	6 (4.1)		
**Employed**,** n (%)**				0.32	0.32
Employed (without sick leave/benefits)	27 (19.3)	29 (20.3)	21 (14.3)		
Employed (with sick leave/disability benefits)	84 (60.0)	88 (61.5)	94 (63.9)		
Unemployed (including 100% disability benefits)	16 (11.4)	12 (8.4)	11 (7.5)		
Retired	7 (5.0)	6 (4.2)	12 (8.2)		
Missing	6 (4.3)	8 (5.6)	9 (6.1)		
**Weekly physical activity level**,** n (%)**				0.81	0.87
No exercise, light activity ≤ 3 h	16 (11.4)	16 (11.2)	14 (9.5)		
No exercise, light activity > 3 h	47 (33.6)	44 (30.8)	54 (36.7)		
Exercise 0–1 h	31 (22.1)	37 (25.9)	37 (25.2)		
Exercise 2–3 h	29 (20.7)	29 (20.3)	26 (17.7)		
Exercise 4 + h	13 (9.3)	10 (7.0)	10 (6.8)		
Missing	4 (2.9)	7 (4.9)	6 (4.1)		
**Smoking**,** n (%)**				0.58	0.55
Never smoker	67 (47.9)	81 (56.6)	78 (53.1)		
Former smoker	58 (41.4)	44 (30.8)	54 (36.7)		
Current smoker	11 (7.9)	11 (7.7)	9 (6.1)		
Missing	4 (2.9)	7 (4.9)	6 (4.1)		
**Drinking alcohol**,** n (%)**				0.03	0.28
No (zero)	54 (38.6)	30 (21.0)	39 (26.5)		
Yes (at least one unit)	81 (57.9)	106 (74.1)	102 (69.4)		
Missing	5 (3.6)	7 (4.9)	6 (4.1)		
**BMI group (kg/m**^**2**^**)**,** n (%)**				0.34	0.56
< 25	59 (42.1)	62 (43.4)	71 (48.3)		
25–29	43 (30.7)	52 (36.4)	45 (30.6)		
≥ 30	32 (22.9)	22 (15.4)	24 (16.3)		
Missing	6 (4.3)	7 (4.9)	7 (4.8)		

^a^Living with children under 18 years.

*CBI* cognitive behavioral therapy intervention, *MBI* mindfulness-based intervention. Data obtained about 1 month after diagnosis (5–8 months prior to randomization) from the Cancer Registry of Norway patient reported outcome measures survey (CRN PROMs survey 1).

*h* hours and *w* week.

The groups were comparable across breast cancer characteristics except for fewer participants in the MBI group with breast cancer stage III (4.9%) than in the control group (13.6%) and CBI (10.9%). Participants in all three groups were enrolled in the trial on average 8 months following diagnosis. Most of the participants had undergone breast conserving therapy, mastectomy or radiation therapy before trial-inclusion. The control group had the lowest proportion with breast conserving therapy at survey response of 18.4%, compared to 25.9% in MBI and 24.3% in CBI (Table 2).

**Table 2:** Breast cancer related background data for participants in all three groups.

Interventions					
Variables	CBI (*N* = 140)	MBI (*N* = 143)	Controls (*N* = 147)	P (CBI vs. Controls)	P (MBI vs. Controls)
**Breast cancer stage**,** n (%)**				0.67	0.27
DCIS	47 (33.6)	52 (36.4)	52 (35.4)		
Stage I	29 (20.7)	33 (23.1)	31 (21.1)		
Stage II	23 (16.4)	26 (18.2)	22 (15.0)		
Stage III	9 (6.4)	7 (4.9)	16 (10.9)		
Missing/neoadjuvantly treated	32 (22.9)	25 (17.5)	26 (17.7)		
**cTNM**,** n (%)**				0.69	0.06
Localised	112 (80.0)	116 (81.1)	108 (73.5)		
Locally advanced	18 (12.9)	10 (7.0)	20 (13.6)		
Missing	10 (7.1)	17 (11.9)	19 (12.9)		
**HER2 status**,** n (%)**				0.39	0.28
Negative	48 (34.3)	47 (32.9)	42 (28.6)		
Positive	49 (35.0)	45 (31.5)	55 (37.4)		
No status^a^	43 (30.7)	51 (35.7)	50 (34.0)		
**ER status**,** n (%)**				0.73	0.68
Negative	61 (43.6)	57 (39.9)	61 (41.5)		
Positive	43 (30.7)	41 (28.7)	39 (26.5)		
No status^a^	36 (25.7)	45 (31.5)	47 (32.0)		
**PR status**,** n (%)**				0.25	0.92
Negative	67 (47.9)	73 (51.0)	74 (50.3)		
Positive	29 (20.7)	21 (14.7)	22 (15.0)		
No status^a^	44 (31.4)	49 (34.3)	51 (34.7)		
**Treatment status at survey response**,** n (%)**				0.63	0.53
Surgery unknown	2 (1.4)	4 (2.8)	5 (3.4)		
BCT	34 (24.3)	37 (25.9)	27 (18.4)		
BCT + radiation therapy	69 (49.3)	63 (44.1)	74 (50.3)		
Masectomy	25 (17.9)	31 (21.7)	30 (20.4)		
Masectomy + radiation therapy	10 (7.1)	8 (5.6)	11 (7.5)		
**Months from diagnosis to response**				0.72	0.85
Mean (SD)	8.2 (1.3)	8.1 (1.2)	8.1 (1.3)		
Median (min-max)	7.8 (6.9–14.5)	7.9 (6.9–11.3)	7.7 (6.9–11.9)		
**Time from diagnosis to response**,** n (%)**				0.35	0.65
< 7 months	12 (8.6)	11 (7.7)	11 (7.5)		
7–8 months	74 (52.9)	77 (53.8)	86 (58.5)		
8–10 months	40 (28.6)	38 (26.6)	30 (20.4)		
>=10 months	14 (10.0)	17 (11.9)	20 (13.6)		

*CBI* cognitive behavioral therapy intervention, *MBI* mindfulness-based intervention, *cTNM* Clinical Stage Group, *HER2* human epidermal growth factor receptor 2, *ER* Estrogen receptor, *PR* progesterone receptors, *BCT* breast conserving therapy. Medical data were obtained from time of diagnosis or at screening about 6–9 months prior to baseline.

^a^1 invasive case without status, the remaining cases have DCIS.

Mean perceived stress levels at baseline were 16.3 (95% CI: 15.0, 17.6) in CBI, 15.5 (95% CI: 14.2, 16.7) in MBI and 15.4 (95% CI: 14.1, 16.7) in controls. Baseline levels of the other secondary outcomes, including HRQoL, coping, depression and anxiety are presented in Table 3. All levels were fairly similar across groups, for instance the HRQoL general health score at baseline was 59.1 (95% CI: 55.7, 62.7) for CBI and 62.0 (95% CI: 58.6, 65.5) for MBI and 62.1 (95% CI:58.8, 65.5) for controls (Table 3).

**Table 3:** Baseline levels and between-group changes in primary and secondary outcomes between those randomized to the cognitive behavioral therapy intervention (CBI) or the mindfulness-based intervention (MBI) compared to the treatment as usual control group.

	CBI (*n* = 140)		MBI (*n* = 143)		Control group (*n* = 147)		CBI vs. control group difference		MBI vs. control group difference	
Primary outcome	M	95% CI	M	95% CI	M	95% CI	MD	95% CI	MD	95% CI
**Perceived stress (PSS-10)**										
Baseline	16.31	15.02, 17.60	15.46	14.19, 16.73	15.40	14.14, 16.67	0.91	−0.90, 2.71	0.06	−1.74, 1.85
6 month follow-up	14.92	13.49, 16.35	14.44	13.07, 15.82	14.68	13.33, 16.03	0.24	−1.71, 2.19	−0.24	−2.17, 1.70
Change adjusted for baseline	−1.23	−2.32, −0.13	−1.09	−2.13, −0.04	−0.81	−1.84, 0.22	−0.42	−1.89, 1.06	−0.28	−1.75, 1.19
***Secondary outcomes***										
**HRQoL (RAND-36)**										
**General health**										
Baseline	59.18	55.69, 62.67	62.05	58.61, 65.48	62.14	58.76, 65.52	−2.96	−7.82, 1.89	−0.10	−4.91, 4.72
6 month follow-up	61.22	57.22, 65.21	64.33	60.27, 68.38	60.36	56.56, 64.17	0.85	−4.57, 6.28	3.97	−1.58, 9.51
Change adjusted for baseline	1.56	−1.48, 4.60	2.50	−0.69, 5.69	−1.54	−4.41, 1.33	3.10	−0.94, 7.14	4.04	−0.18, 8.26
**Physical functioning**										
Baseline	74.77	71.32, 78.22	78.74	75.32, 82.16	77.67	74.32, 81.02	−2.90	−7.71, 1.90	1.07	−3.70, 5.85
6 month follow-up	77.52	73.76, 81.27	81.06	77.27, 84.84	78.29	74.71, 81.88	−0.78	−6.00, 4.45	2.76	−2.44, 7.96
Change adjusted for baseline	2.21	−0.55, 4.97	2.70	−0.10, 5.51	0.76	−1.82, 3.35	1.44	−2.37, 5.26	1.94	−1.84, 5.72
**Role-physical**										
Baseline	32.30	25.13, 39.48	36.87	29.81, 43.93	34.51	27.60, 41.43	−2.21	−12.17, 7.75	2.36	−7.53, 12.25
6 month follow-up	45.14	37.01, 53.27	46.78	38.29, 55.28	47.69	39.67, 55.71	−2.55	−13.87, 8.78	−0.91	−12.83, 11.01
Change adjusted for baseline	11.95	4.99, 18.91	10.81	3.39, 18.22	13.15	6.30, 20.01	−1.20	−10.82, 8.41	−2.34	−12.71, 8.02
**Role-emotional**										
Baseline	56.97	49.60, 64.34	62.40	55.16, 69.64	61.30	54.22, 68.38	−4.33	−14.57, 5.91	1.10	−9.03, 11.23
6 month follow-up	63.84	55.39, 72.29	67.52	59.28, 75.76	71.00	63.27, 78.73	−7.16	−18.68, 4.36	−3.48	−14.78, 7.82
Change adjusted for baseline	4.87	−3.08, 12.83	6.42	−1.35, 14.19	10.33	3.08, 17.59	−5.46	−16.24, 5.31	−3.91	−14.48, 6.66
**Vitality**										
Baseline	43.42	39.46, 47.39	46.30	42.41, 50.19	45.40	41.56, 49.23	−1.97	−7.49, 3.54	0.90	−4.56, 6.37
6 month follow-up	48.92	44.48, 53.35	51.03	46.65, 55.40	47.40	43.26, 51.53	1.52	−4.52, 7.56	3.63	−2.47, 9.73
Change adjusted for baseline	5.06	1.58, 8.53	5.06	1.68, 8.44	2.09	−0.96, 5.14	2.97	−1.59, 7.52	2.97	−1.67, 7.61
**Mental health**										
Baseline	69.21	66.12, 72.29	71.65	68.62, 74.69	71.22	68.22, 74.21	−2.01	−6.31, 2.28	0.43	−3.83, 4.70
6 month follow-up	72.35	69.25, 75.45	73.99	70.92, 77.05	74.14	71.17, 77.10	−1.79	−6.02, 2.44	−0.15	−4.39, 4.09
Change adjusted for baseline	2.63	0.26, 5.01	2.66	0.28, 5.03	3.09	0.84, 5.34	−0.46	−3.62, 2.71	−0.43	−3.62, 2.76
**Social functioning**										
Baseline	61.57	57.17, 65.96	67.45	63.12, 71.78	64.61	60.34, 68.88	−3.04	−9.16, 3.08	2.84	−3.23, 8.92
6 month follow-up	68.92	64.34, 73.51	72.88	68.19, 77.56	70.99	66.59, 75.39	−2.07	−8.31, 4.18	1.89	−4.52, 8.30
Change adjusted for baseline	6.29	2.62, 9.96	6.46	2.58, 10.33	6.40	2.89, 9.90	−0.11	−4.99, 4.78	0.06	−5.07, 5.19
**Bodily pain**										
Baseline	60.04	55.92, 64.16	63.60	59.55, 67.64	62.68	58.70, 66.66	−2.64	−8.37, 3.09	0.91	−4.76, 6.59
6 month follow-up	63.66	58.69, 68.63	64.79	59.97, 69.61	63.81	59.10, 68.51	−0.14	−7.05, 6.77	0.99	−5.75, 7.72
Change adjusted for baseline	2.95	−1.20, 7.10	1.67	−2.34, 5.67	1.30	−2.55, 5.16	1.65	−4.05, 7.35	0.37	−5.20, 5.93
**Mindfulness (FFMQ-15)**										
Baseline	12.34	11.86, 12.81	12.88	12.41, 13.35	12.78	12.32, 13.24	−0.45	−1.11, 0.22	0.10	−0.56, 0.75
6 month follow-up	12.77	12.24, 13.30	13.41	12.89, 13.92	12.84	12.33, 13.36	−0.08	−0.82, 0.66	0.56	−0.16, 1.29
Change adjusted for baseline	0.35	−0.05, 0.75	0.58	0.20, 0.97	0.09	−0.30, 0.48	0.26	−0.30, 0.82	0.49	−0.05, 1.03
**TOMCATS**										
**Coping**										
Baseline	3.11	3.02, 3.20	3.17	3.08, 3.26	3.04	2.95, 3.13	0.07	−0.06, 0.20	0.14	0.01, 0.26
6 month follow-up	3.01	2.91, 3.12	3.17	3.06, 3.27	3.10	3.00, 3.20	−0.09	−0.23, 0.06	0.07	−0.08, 0.22
Change adjusted for baseline	−0.09	−0.19, 0.00	0.03	−0.06, 0.13	0.02	−0.07, 0.11	−0.12	−0.25, 0.02	0.01	−0.12, 0.15
**Helplessness**										
Baseline	2.07	1.95, 2.19	1.95	1.83, 2.07	1.99	1.87, 2.11	0.08	−0.09, 0.25	−0.04	−0.21, 0.13
6 month follow-up	2.06	1.93, 2.20	1.97	1.83, 2.11	1.97	1.84, 2.10	0.09	−0.10, 0.29	−0.00	−0.19, 0.19
Change adjusted for baseline	0.02	−0.10, 0.13	0.00	−0.12, 0.12	−0.02	−0.13, 0.09	0.04	−0.12, 0.20	0.03	−0.14, 0.19
**Hopelessness**										
Baseline	1.67	1.56, 1.78	1.56	1.45, 1.67	1.56	1.46, 1.67	0.10	−0.05, 0.26	−0.00	−0.16, 0.15
6 month follow-up	1.58	1.47, 1.70	1.52	1.40, 1.63	1.59	1.47, 1.71	−0.00	−0.17, 0.17	−0.07	−0.24, 0.09
Change adjusted for baseline	−0.05	−0.15, 0.05	−0.06	−0.16, 0.04	0.01	−0.09, 0.12	−0.06	−0.21, 0.08	−0.07	−0.21, 0.07
**Global Fatigue (CFQ-11)**										
Baseline	20.38	19.43, 21.33	19.50	18.57, 20.44	20.00	19.08, 20.92	0.38	−0.94, 1.70	−0.50	−1.81, 0.81
6 month follow-up	18.77	17.69, 19.85	17.35	16.21, 18.49	18.57	17.52, 19.62	0.20	−1.31, 1.70	−1.22	−2.77, 0.33
Change adjusted for baseline	−1.51	−2.33, −0.69	−2.27	−3.18, −1.35	−1.42	−2.21, −0.64	−0.09	−1.24, 1.06	−0.84	−2.06, 0.37
**Anxiety and depression (PHQ-4)**										
Baseline	3.40	2.92, 3.87	3.04	2.57, 3.50	3.18	2.72, 3.64	0.22	−0.44, 0.88	−0.14	−0.80, 0.51
6 month follow-up	3.08	2.62, 3.55	2.72	2.26, 3.18	2.84	2.39, 3.29	0.24	−0.40, 0.89	−0.12	−0.76, 0.52
Change adjusted for baseline	−0.24	−0.59, 0.11	−0.38	−0.74, −0.02	−0.35	−0.69, −0.00	0.11	−0.40, 0.61	−0.03	−0.52, 0.46
**Sleep (hours)**										
Baseline	7.04	6.81, 7.27	7.09	6.87, 7.32	7.16	6.94, 7.38	−0.12	−0.43, 0.20	−0.07	−0.38, 0.25
6 month follow-up	7.33	6.80, 7.85	7.20	6.80, 7.60	7.22	6.84, 7.61	0.10	−0.55, 0.75	−0.02	−0.58, 0.54
Change adjusted for baseline	0.27	−0.23, 0.77	0.11	−0.25, 0.47	0.07	−0.27, 0.42	0.20	−0.40, 0.79	0.03	−0.47, 0.53
**Sleep (summary score)**										
Baseline	19.08	17.20, 20.95	17.73	15.88, 19.57	17.62	15.81, 19.44	1.45	−1.16, 4.06	0.10	−2.48, 2.69
6 month follow-up	17.48	15.42, 19.53	16.86	14.88, 18.84	16.20	14.20, 18.19	1.28	−1.59, 4.14	0.66	−2.17, 3.50
Change adjusted for baseline	−1.33	−2.91, 0.26	−0.98	−2.48, 0.52	−1.57	−3.10, −0.04	0.24	−1.95, 2.44	0.59	−1.59, 2.77

*M* mean, *MD* mean difference, *PSS* perceived stress scale, *HRQoL* Health- related quality of life, *RAND-36* RAND corporation 36-item Short Form health Survey, *FFMQ* Five Facet Mindfulness Questionnaire, *TOMCATS* Theoretically Originated Measure of the Cognitive Activation Theory of Stress, *CFQ-11* Chalder fatigues questionnaire 11 items, *PHQ* Patient health questionnaire.

### Between group differences

After 6 months of access to the interventions, there was no statistically significant difference in perceived stress levels adjusted for baseline levels between either of the intervention groups and the control group. Similarly, there were no significant differences in HRQoL subscales, levels of anxiety and depression, mindfulness, fatigue, coping or sleep between the three groups. When combining the intervention groups, the HRQoL general health score improved significantly more in the intervention groups combined vs. the control group (3.58 [95% CI: 0.06–7.09]) (Table 3).

All outcomes yielded similar results in the complete case analysis (Supplementary file 5). Furthermore, we additionally adjusted for a-priori defined potential confounders and the results were fairly similar to the main results presented in Table 3 (Supplementary file 6).

### Subgroup (interaction) analyses

#### Perceived stress level

**Fig. 2:**
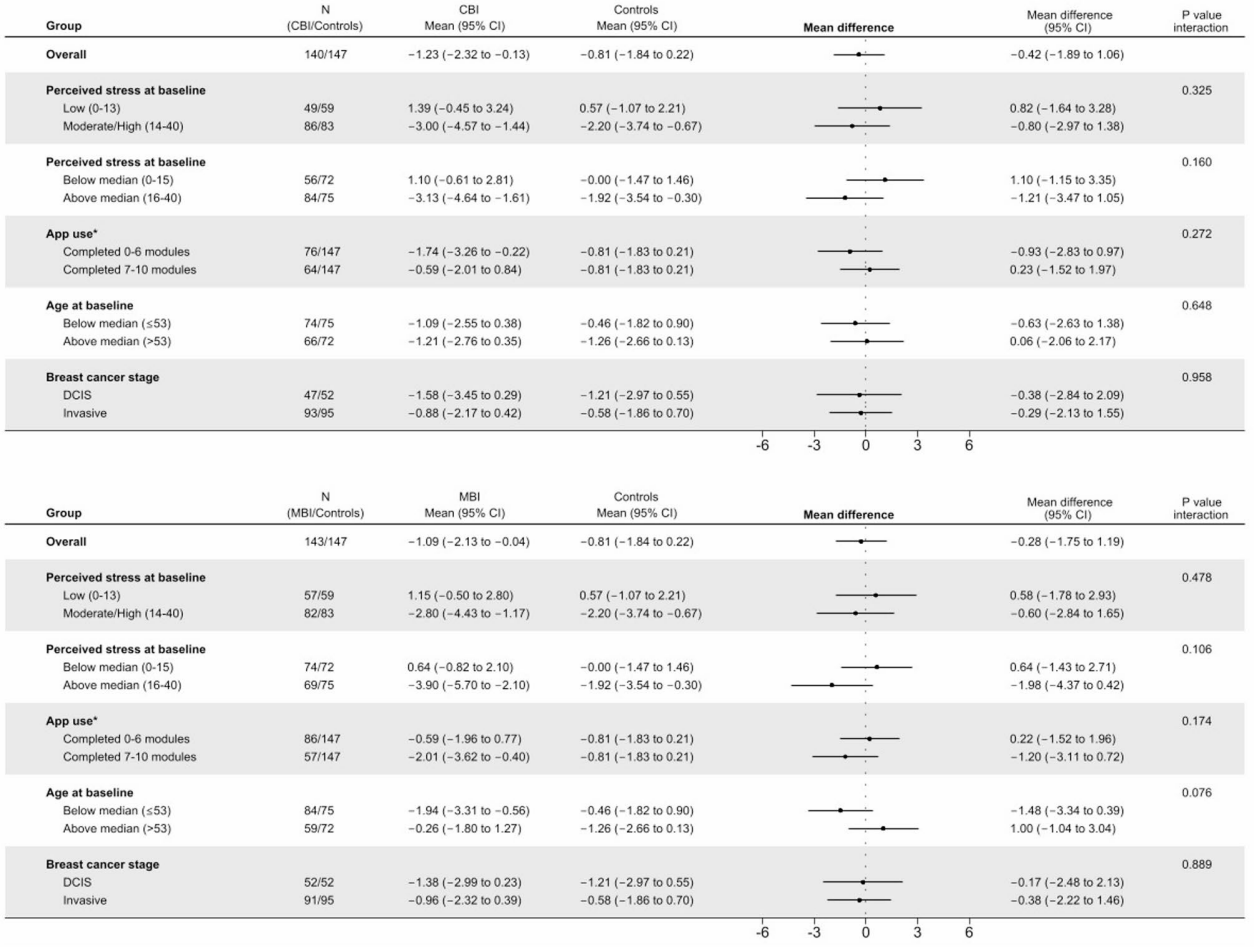
Results from subgroup analyses of mean difference in perceived stress level at baseline (low vs. moderate stress level and below vs. above median stress level) and app use (those who completed 0–6 vs. 7–10 modules), age groups (below vs. above median age) and stage (DCIS vs. invasive) in CBI vs. controls (upper panel), and MBI vs. controls (lower panel). *CBI* cognitive behavioral therapy intervention, *MBI* mindfulness-based intervention.

Individuals with highest perceived stress level at baseline, especially in the MBI group, had a somewhat larger reduction in perceived stress after 6 months compared to individuals with lower mean levels at baseline, although this interaction was not statistically significant. Young participants (≤ 53 years) in the MBI group had a larger reduction in perceived stress than young controls, while the older MBI participants (> 53 years) had less stress reduction than older controls (p for interaction 0.076) (Fig. 2).

#### Use of the intervention (app use)

In total, 43% completed at least 7 of the 10 modules of any intervention (i.e., defined as completers in accordance with Børøsund and colleagues^35^ whereas 19% completed all 10 modules. Fourteen (5%) of the 238 participants did not appear to have used the app at all. For MBI, completers had a higher reduction of perceived stress level than those completing fewer modules, but this interaction was not statistically significant and was not observed for CBI (Fig. 2).

Participants assigned to MBI had a higher median (min-max) frequency of use of 8 (1–144) active days, compared to 5 (1–63) active days in CBI, whereas participants assigned to CBI completed more modules (6 vs. 4) and had a higher number of completers (46% vs. 40%) (Table 4).

**Table 4:** Intervention (app) use among participants randomized to the CBI and MBI interventions.

Variables	CBI (*n* = 140)	MBI (*n* = 143)	Intervention groups combined (*n* = 283)	*P* ^a^
**Number of active days of use**				0.03
Mean (SD)	9.7 (12.3)	13.7 (18.4)	11.8 (15.8)	
Median (min-max)	5.0 (1.0–63.0)	8.0 (1.0–144.0.0.0)	7.0 (1.0–144.0.0.0)	
**Days from first to last use**				0.75
Mean (SD)	97.2 (59.0)	103.7 (55.6)	100.5 (57.3)	
Median (min-max)	86.5 (1.0–182.0.0.0)	100.0 (1.0–185.0.0.0)	97.0 (1.0–185.0.0.0)	
**Number of completed modules**				0.34
Mean (SD)	5.4 (3.7)	5.0 (3.7)	5.2 (3.7)	
Median (min-max)	6.0 (0.0–10.0)	4.0 (0.0–10.0)	5.0 (0.0–10.0)	
**Completed 7–10 modules**				0.32
n (%)	64 (45.7)	57 (39.9)	121 (42.8)	
**Completed all modules**				0.59
n (%)	29 (20.7)	26 (18.2)	55 (19.4)	
**No registered app use**				0.56
n (%)	8 (5.7)	6 (4.2)	14 (4.9)	

*CBI* Cognitive Behavioral Therapy Intervention, *MBI* mindfulness-based intervention.

^a^P for MBI vs. CBI.

## Discussion

The current CABC trial with early breast cancer survivors was initiated to enable comparison of the effects of different digital stress-management interventions (cognitive behavioral [i.e., CBI] or mindfulness-based content [i.e., MBI]) compared to usual-care controls. Results after 6-months of intervention access showed no statistically significant differences between either intervention group and the control group in terms of the selected outcome measures.

The lack of significant findings when comparing the CBI and MBI interventions and the control group was somewhat surprising, given that access to the original StressProffen intervention program (combining CBI and MBI content) previously had been associated with reduced perceived stress, anxiety and depression, as well as improved self-regulatory fatigue and HRQoL for cancer survivors after 6 and 12 months^35^. There are several possible explanations for the apparent lack of intervention effects after 6 months in the CABC trial. First, baseline perceived stress levels were on average low for all groups, corresponding to those of healthy female populations at similar ages^54–56^. This creates a floor effect and leaves less room for further reductions^32,57,58^, which was also evident in the subgroup analysis where those with the highest perceived stress level at baseline displayed larger reductions in perceived stress levels than those with lower baseline stress level. However, these findings were non-significant. This notion is supported by other digital stress-management interventions, where a certain level of stress or distress has been part of the inclusion criteria^59,60^. Nevertheless, there are several methods to assess stress, and it is possible that other effects could have been detected if different outcome measures were applied^60^.

Second, the digital download-only delivery method employed in the current study may have acted as a barrier to use and hence reduced the effect of the interventions. The original StressProffen digital intervention was developed based on evidence (i.e., evidence-based CBT and Mindfulness interventions), with extensive user-involvement^33^, as were the current CBI and MBI interventions^37^. Adherence is, however, a significant challenge to the use and impact of digital health interventions^61,62^, and appears to have impacted the current study accordingly. In total 43% of participants were defined as intervention completers^35^ at 6 months in this study, and the intervention use in the CABC trial therefore falls below an average of 50% adherence in eHealth interventions^61^. In comparison, completion rates in the original StressProffen trials were 58% at 3 months^34^ and 68% at 12 months^35^. There was also a lower frequency of app use in the CABC trial compared to the original StressProffen trial(s), with participants using the apps a median of 7 times (range 1–144) in this study, and 17.5 times (range 3–170) in the StressProffen trial^35,37^. Intended benefit from interventions can only be achieved through actual use of the interventions, and if digital download-only, as used in this trial, introduces a barrier to use, this will also indicate a barrier to intended effect^58^. It is possible that a similar in-person introduction session as used in the original StressProffen trial, or perhaps a less resource requiring introduction and follow-up via secure video link, could have improved intervention adherence and subsequently intervention effects.

Third, “one-size does not fit all”. While in-person interventions allow for tailoring depending on setting, options for individual tailoring in digital interventions are still limited. For practical purposes, the current study delivered both CBI and MBI interventions as standard protocols, without tailoring based on preference or other possibly relevant mechanisms or dysregulations, potentially curbing intervention impact. The lack of pre-treatment screening for individual preference, stress and distress-levels as well as survivorship stage, could perhaps partly explain why the findings between the two tested interventions were not significant. For example, there are indications that MBIs may be particularly effective during the early post-treatment transition period (3–12 months post-treatment) when somatic hypervigilance, uncertainty, and “in-between” feelings peak^63^. Accordingly, MBI’s acceptance strategies may not have had appropriate existential content to work with in periods of their survivorship when they have plenty of other needs and actions to fill their awareness and time^64^.

The non-significant differences in outcomes between intervention groups could hence reflect that both approaches (i.e., CBI and MBI), when appropriately matched to patient needs and optimal timing windows, may be effective. Identifying what works for whom, when and where is likely essential in such psychosocial treatment settings^65^. Providing options for tailoring digital interventions such as StressProffen might be vital, simulating guidance, facilitating engagement and inspiring program adherence and subsequent effect^66,67^. In line with this, since anxiety and depression may peak around the time of diagnosis, whereas sleep disturbances, fatigue and pain may occur at later stages or persist long-term^68^, there may have been potential intervention effects prior to the 6-month assessment that were not captured. Similarly, potential interventions effects after 6 months have yet to be evaluated. It is also possible the CABC interventions could have been more effective if used during active cancer treatment^3,69^. Introducing new activities such as psychosocial interventions during the early days following diagnosis can, however, be challenging, and in the current study, avoiding interference with surgery or medical breast cancer treatment was considered important^70^.

Another factor potentially impacting motivation for use could have been the recruitment methods used. In the CABC trial, only responders of the digital, ongoing CRN PROMs survey sent to all breast cancer patients in Norway^39^ were invited, while participants in the original StressProffen trials were broadly recruited through social media (i.e., self-motivation) and local cancer treatment institutions (i.e., through familiar hospital settings/providers)^35^.

When combining the intervention groups, improved general health on the HRQoL scale was detected in favor of the intervention groups compared to controls, within the range of a minimally important difference^44,71,72^ and in line with other digital intervention studies^58^. However, the effect was rather small, and thus of limited clinical relevance.

We observed some interesting patterns in the subgroup analysis. The intervention effect went in opposite directions for younger and older MBI participants. This may indicate that MBI appeal more to the younger cancer population^73^. Furthermore, there were some differences in terms of app use, with participants in the CBI group completing the most modules, but participants in the MBI group using the app more frequently. Given the low effect sizes and the lack of other types of significant findings, these patterns should nevertheless be interpreted with caution.

### Strengths and limitations

The CABC trial has several strengths. First, with 430 breast cancer patients, it is one of the largest sampled population-based RCTs of digital stress-management interventions^74^, with robust trial design and a planned long-term follow-up with survey data to 36 months after diagnosis. The outcome measure completion rate, ≥ 71% at 6-months, was only slightly lower than in the original StressProffen (77%)^35^, and acceptable in comparison to other eHealth interventions^58^. The study reports data on local treatments but lacks information on systemic treatments. The trial also has other limitations. First, there is a lack of diversity in participant demographic characteristics^8^, as participants were predominantly highly educated, and also, according to national Norwegian data on the same sample, predominantly Caucasian/White. Furthermore, the sample does not reflect the total breast cancer population in Norway as we, in order to avoid overlap with ongoing treatment trials in Norway only included selected subtypes of breast cancer. Second, as this trial relied on participation through the ongoing CRN PROMs survey (i.e., response to written invitations), the sample was in many ways self-selected. Thirdly, the study did not control for activities within the control group. Participants in the control group may have participated in other types of stress-management activities during the study period without the research team being able to control for such activities at this timepoint.

## Future directions

Acquiring psychoeducational knowledge and incorporating such knowledge into daily use and practice takes time. Future studies should therefore explore whether longer term access to digital health interventions such as CBI and MBI can yield more significant indications of effect. Post-study qualitative interviews with participants to understand pattern of use or non-use could also shed light on what works for whom and when in such settings. As the original StressProffen intervention^35^ was not included in the current trial, future research might also consider comparing the original version (i.e., primarily CBT-based but with aspects of mindfulness) with the two developed CBI and MBI versions, to determine whether a combination of CBI and MBI is in fact superior to both interventions separately. Intervention preferences may also play a role, and future research should explore whether having the option to choose between the two types of stress-management interventions presented in this trial may enhance motivation, use and subsequently potential effects^65,75–77^. Incorporating aspects from persuasive design, motivational interviewing, behavior change theories and techniques might also facilitate engagement and adherence to eHealth interventions^66,78–81^. The StressProffen app contains some individualized options (e.g., option to read or listen, and mark own favorites), but not other potentially adherence-promoting features such as gamification and option to connect to a community, or join group sessions^69,82^. Future research may explore additional options for improving adherence to digital interventions, potentially also incorporating follow-up contact either by phone, chat or video at later timepoints than conducted in the current study.

## Conclusion

In the CABC trial, women with breast cancer receiving either a digital stress-management intervention with cognitive behavioral or mindfulness-based content did not show significantly greater improvements in perceived stress, anxiety, depression, fatigue, HRQoL, mindfulness, coping, or sleep after 6 months of intervention access compared to usual-care controls. Perceived stress at baseline was however low in all groups, which may have impacted the outcome. This was a digital download-only intervention trial, and further research is needed to determine the best way to deliver digital health interventions to facilitate use and subsequent intended effect. Timing of delivery during the cancer trajectory to achieve impact, types of stress-management interventions, and what works best for whom and when, should also be explored.

## Supplementary Information

Below is the link to the electronic supplementary material.

Supplementary Material 1

Supplementary Material 2

Supplementary Material 3

## References

[1] Siegel, R. L. et al. Cancer statistics, 2022. *CA Cancer J. Clin.* **72** (1), 7–33 (2022). 35020204 10.3322/caac.21708

[2] The Cancer Registry of Norway. *Cancer in Norway 2023- Cancer incidence, mortality, Survival and Prevalence in Norway* (Oslo, 2024).

[3] Antoni, M. H., Moreno, P. I. & Penedo, F. J. Stress management interventions to facilitate psychological and physiological adaptation and optimal health outcomes in cancer patients and survivors. *Annu. Rev. Psychol.* **74**, 423–455 (2023). 35961041 10.1146/annurev-psych-030122-124119 PMC10358426

[4] Stanton, A. L., Rowland, J. H. & Ganz, P. A. Life after diagnosis and treatment of cancer in adulthood: contributions from psychosocial oncology research. *Am. Psychol.* **70** (2), 159–174 (2015). 25730722 10.1037/a0037875

[5] Andersen, B. L., Kiecolt-Glaser, J. K. & Glaser, R. A biobehavioral model of cancer stress and disease course. *Am. Psychol.* **49** (5), 389–404 (1994). 8024167 10.1037//0003-066x.49.5.389 PMC2719972

[6] Antoni, M. H. & Dhabhar, F. S. The impact of psychosocial stress and stress management on immune responses in patients with cancer. *Cancer* **125** (9), 1417–1431 (2019). 30768779 10.1002/cncr.31943 PMC6467795

[7] Singer, S. Psychosocial impact of cancer. *Recent. Results Cancer Res.* **210**, 1–11 (2018). 28924676 10.1007/978-3-319-64310-6_1

[8] Carlson, L. E. Psychosocial and integrative oncology: interventions across the disease trajectory. *Annu. Rev. Psychol.* **74**, 457–487 (2023). 36104001 10.1146/annurev-psych-032620-031757

[9] Heidary, Z. et al. Quality of life in breast cancer patients: A systematic review of the qualitative studies. *Cancer Control*. **30**, 10732748231168318 (2023). 37082898 10.1177/10732748231168318 PMC10236425

[10] Javan Biparva, A. et al. Global Quality of Life in Breast Cancer: Systematic Review and meta-analysis. BMJ Support Palliat Care, 2022. 10.1136/bmjspcare-2022-003642 PMC10850719 35710706

[11] Mokhtari-Hessari, P. & Montazeri, A. Correction to: Health-related quality of life in breast cancer patients: review of reviews from 2008 to 2018. *Health Qual. Life Outcomes*. **20** (1), 35 (2022). 35216596 10.1186/s12955-022-01942-w PMC8881806

[12] Ursin, H. & Eriksen, H. R. The cognitive activation theory of stress. *Psychoneuroendocrinology* **29** (5), 567–592 (2004). 15041082 10.1016/S0306-4530(03)00091-X

[13] Aricò, D., Raggi, A. & Ferri, R. Cognitive behavioral therapy for insomnia in breast cancer survivors: A review of the literature. *Front. Psychol.* **7**, 1162 (2016). 27536265 10.3389/fpsyg.2016.01162 PMC4971442

[14] Naaman, S. C. et al. Status of psychological trials in breast cancer patients: a report of three meta-analyses. *Psychiatry* **72** (1), 50–69 (2009). 19366294 10.1521/psyc.2009.72.1.50

[15] Antoni, M. H. et al. How stress management improves quality of life after treatment for breast cancer. *J. Consult Clin. Psychol.* **74** (6), 1143–1152 (2006). 17154743 10.1037/0022-006X.74.6.1152 PMC5752106

[16] Haller, H. et al. Mindfulness-based interventions for women with breast cancer: an updated systematic review and meta-analysis. *Acta Oncol.* **56** (12), 1665–1676 (2017). 28686520 10.1080/0284186X.2017.1342862

[17] Zhang, M. et al. Effects of cognitive behavioral therapy on quality of life and stress for breast cancer survivors: a meta-analysis. *Minerva Med.* **108** (1), 84–93 (2017). 27635602 10.23736/S0026-4806.16.04528-6

[18] Andersen, B. L. et al. Management of anxiety and depression in adult survivors of cancer: ASCO guideline update. *J. Clin. Oncol.* **41** (18), 3426–3453 (2023). 37075262 10.1200/JCO.23.00293

[19] Getu, M. A. et al. The effect of cognitive behavioral therapy on the quality of life of breast cancer patients: a systematic review and meta-analysis of randomized controlled trials. *Qual. Life Res.* **30** (2), 367–384 (2021). 33068239 10.1007/s11136-020-02665-5

[20] Guarino, A. et al. The effectiveness of psychological treatments in women with breast cancer: A systematic review and meta-analysis. *J. Clin. Med.* **9**, 1 (2020). 10.3390/jcm9010209 PMC7019270 31940942

[21] Schell, L. K. et al. Mindfulness-based stress reduction for women diagnosed with breast cancer. *Cochrane Database Syst. Rev.* **3** (3), pCd011518 (2019). 10.1002/14651858.CD011518.pub2 PMC6436161 30916356

[22] Ding, X. et al. A systematic review and meta-analysis of interventions to reduce perceived stress in breast cancer patients. *Complement. Ther. Clin. Pract.* **54**, 101803 (2024). 38159534 10.1016/j.ctcp.2023.101803

[23] Chang, Y. C. et al. Immediate impact of Mindfulness-Based cognitive therapy (MBCT) among women with breast cancer: a systematic review and meta-analysis. *BMC Womens Health*. **23** (1), 331 (2023). 37349700 10.1186/s12905-023-02486-x PMC10288664

[24] Carlson, L. E. Mindfulness-based interventions for coping with cancer. *Ann. N Y Acad. Sci.* **1373** (1), 5–12 (2016). 26963792 10.1111/nyas.13029

[25] Hölzel, B. K. et al. Stress reduction correlates with structural changes in the amygdala. *Soc. Cogn. Affect. Neurosci.* **5** (1), 11–17 (2010). 19776221 10.1093/scan/nsp034 PMC2840837

[26] Zeichner, S. B. et al. Cognitive behavioral therapy for Insomnia, Mindfulness, and yoga in patients with breast cancer with sleep disturbance: A literature review. *Breast Cancer (Auckl)*. **11**, 1178223417745564 (2017). 29434470 10.1177/1178223417745564 PMC5802619

[27] Hulbert-Williams, N. J., Beatty, L. & Dhillon, H. M. Psychological support for patients with cancer: evidence review and suggestions for future directions. *Curr. Opin. Support Palliat. Care*. **12** (3), 276–292 (2018). 30074924 10.1097/SPC.0000000000000360

[28] Olsson Möller, U. et al. A comprehensive approach to rehabilitation interventions following breast cancer treatment - a systematic review of systematic reviews. *BMC Cancer*. **19** (1), 472 (2019). 31109309 10.1186/s12885-019-5648-7 PMC6528312

[29] Garland, S. N., Mahon, K. & Irwin, M. R. Integrative approaches for sleep health in cancer survivors. *Cancer J.* **25** (5), 337–342 (2019). 31567461 10.1097/PPO.0000000000000398

[30] Leslie, M. et al. Web-Based psychological interventions for people living with and beyond cancer: Meta-Review of what works and what does not for maximizing Recruitment, Engagement, and efficacy. *JMIR Cancer*. **8** (3), e36255 (2022). 35802418 10.2196/36255 PMC9308073

[31] Huang, Y. et al. Effectiveness of internet-based support interventions on patients with breast cancer: a systematic review and narrative synthesis. *BMJ Open.* **12** (5), e057664 (2022). 35641011 10.1136/bmjopen-2021-057664 PMC9157353

[32] Kirsch, E. P. et al. Digital health platforms for breast cancer care: a scoping review. *J. Clin. Med.* **13**, 7 (2024). 10.3390/jcm13071937 PMC11012307 38610702

[33] Børøsund, E. et al. A stress management app intervention for cancer survivors: Design, Development, and usability testing. *JMIR Form. Res.* **2** (2), e19 (2018). 30684438 10.2196/formative.9954 PMC6334690

[34] Børøsund, E. et al. Results from a randomized controlled trial testing StressProffen; an application-based stress-management intervention for cancer survivors. *Cancer Med.* **9** (11), 3775–3785 (2020). 32243717 10.1002/cam4.3000 PMC7286452

[35] Børøsund, E. et al. Digital stress management in cancer: testing stressproffen in a 12-month randomized controlled trial. *Cancer* **128** (7), 1503–1512 (2022). 34855212 10.1002/cncr.34046

[36] Børøsund, E. et al. Pilot testing an app-based stress management intervention for cancer survivors. *Transl Behav. Med.* **10** (3), 770–780 (2020). 31330023 10.1093/tbm/ibz062 PMC7413188

[37] Børøsund, E. et al. Digital cognitive Behavioral- and Mindfulness-Based Stress-Management interventions for survivors of breast cancer: development study. *JMIR Form. Res.* **7**, e48719 (2023). 37725424 10.2196/48719 PMC10548331

[38] Svendsen, K. et al. Coping after breast cancer: protocol for a randomized controlled trial of stress management eHealth interventions. *JMIR Res. Protoc.* **12**, e47195 (2023). 37103493 10.2196/47195 PMC10248777

[39] Gjelsvik, Y., Johannesen, T. B., Ursin, G. & Mykelbust, T. Å. A nationwide prospective collection of patient reported outcomes in the Cancer Registry of Norway. Norsk Epidemiologi, (2022).

[40] Chan, J. Exploring digital health care: eHealth, mHealth, and librarian opportunities. *J. Med. Libr. Assoc.* **109** (3), 376–381 (2021). 34629965 10.5195/jmla.2021.1180 PMC8485950

[41] Cohen, S., Kamarck, T. & Mermelstein, R. A global measure of perceived stress. *J. Health Soc. Behav.* **24** (4), 385–396 (1983). 6668417

[42] Kroenke, K. et al. An Ultra-Brief screening scale for anxiety and depression: the PHQ–4. *Psychosomatics* **50** (6), 613–621 (2009). 19996233 10.1176/appi.psy.50.6.613

[43] Chalder, T. et al. Development of a fatigue scale. *J. Psychosom. Res.* **37** (2), 147–153 (1993). 8463991 10.1016/0022-3999(93)90081-p

[44] Hays, R. D. & Morales, L. S. The RAND-36 measure of health-related quality of life. *Ann. Med.* **33** (5), 350–357 (2001). 11491194 10.3109/07853890109002089

[45] Baer, R. A. et al. Using self-report assessment methods to explore facets of mindfulness. *Assessment* **13** (1), 27–45 (2006). 16443717 10.1177/1073191105283504

[46] Bohlmeijer, E. et al. Psychometric properties of the five facet mindfulness questionnaire in depressed adults and development of a short form. *Assessment* **18** (3), 308–320 (2011). 21586480 10.1177/1073191111408231

[47] Odeen, M. et al. Expectancies, socioeconomic status, and self-rated health: use of the simplified TOMCATS questionnaire. *Int. J. Behav. Med.* **20** (2), 242–251 (2013). 22294319 10.1007/s12529-012-9221-x PMC3641306

[48] Natvik, S. et al. Personality factors related to shift work tolerance in two- and three-shift workers. *Appl. Ergon.* **42** (5), 719–724 (2011). 21172694 10.1016/j.apergo.2010.11.006

[49] Børøsund, E. et al. Digital stress management in cancer: testing stressproffen in a 12-month randomized controlled trial. *Cancer* (2021). 10.1002/cncr.34046 34855212

[50] Aaronson, N. K. et al. The European organization for research and treatment of cancer QLQ-C30: a quality-of-life instrument for use in international clinical trials in oncology. *J. Natl. Cancer Inst.* **85** (5), 365–376 (1993). 8433390 10.1093/jnci/85.5.365

[51] Sullivan, T. R. et al. Should multiple imputation be the method of choice for handling missing data in randomized trials? *Stat. Methods Med. Res.* **27** (9), 2610–2626 (2018). 28034175 10.1177/0962280216683570 PMC5393436

[52] van Buuren, S. *Flexible imputation of missing data*. 2. ed. Interdisciplinary Statistics Series 2018, Florida: Chapman & Hall / CRC Press.

[53] Nguyen, C. D., Carlin, J. B. & Lee, K. J. Model checking in multiple imputation: an overview and case study. *Emerg. Themes Epidemiol.* **14** (1), 8 (2017). 28852415 10.1186/s12982-017-0062-6 PMC5569512

[54] Cohen, S. & Janicki-Deverts, D. Who’s stressed? Distributions of psychological stress in the united States in probability samples from 1983, 2006, and 20091. *J. Appl. Soc. Psychol.* **42** (6), 1320–1334 (2012).

[55] Nordin, M. & Nordin, S. Psychometric evaluation and normative data of the Swedish version of the 10-item perceived stress scale. *Scand. J. Psychol.* **54** (6), 502–507 (2013). 24118069 10.1111/sjop.12071

[56] Klein, E. M. et al. The German version of the perceived stress Scale - psychometric characteristics in a representative German community sample. *BMC Psychiatry*. **16**, 159 (2016). 27216151 10.1186/s12888-016-0875-9 PMC4877813

[57] Stanton, A. L. How and for whom? Asking questions about the utility of psychosocial interventions for individuals diagnosed with cancer. *J. Clin. Oncol.* **23** (22), 4818–4820 (2005). 15939929 10.1200/JCO.2005.01.913

[58] Seiler, A. et al. eHealth and mHealth interventions in the treatment of fatigued cancer survivors: A systematic review and meta-analysis. *Psychooncology* **26** (9), 1239–1253 (2017). 28665554 10.1002/pon.4489

[59] Nixon, P. et al. The efficacy of a Web-Based stress management intervention for employees experiencing adverse working conditions and occupational Self-efficacy as a mediator: randomized controlled trial. *J. Med. Internet Res.* **24** (10), e40488 (2022). 36264607 10.2196/40488 PMC9634524

[60] Mengin, A. C. et al. Efficacy of the my health too online cognitive behavioral therapy program for healthcare workers during the COVID-19 pandemic: A randomized controlled trial. *Internet Interv*. **36**, 100736 (2024). 38617386 10.1016/j.invent.2024.100736 PMC11015127

[61] Singleton, A. C. et al. Electronic health interventions for patients with breast cancer: systematic review and Meta-Analyses. *J. Clin. Oncol.* **40** (20), 2257–2270 (2022). 35500200 10.1200/JCO.21.01171 PMC9273371

[62] Meyerowitz-Katz, G. et al. Rates of attrition and dropout in App-Based interventions for chronic disease: systematic review and Meta-Analysis. *J. Med. Internet Res.* **22** (9), e20283 (2020). 32990635 10.2196/20283 PMC7556375

[63] Knobf, M. T. The transition experience to breast cancer survivorship. *Semin Oncol. Nurs.* **31** (2), 178–182 (2015). 25951747 10.1016/j.soncn.2015.02.006

[64] Ross, L. W., Townsend, J. S. & Rohan, E. A. Still lost in transition? Perspectives of ongoing cancer survivorship care needs from comprehensive cancer control programs, survivors, and health care providers. *Int. J. Environ. Res. Public. Health* **19**, 5 (2022). 10.3390/ijerph19053037 PMC8910165 35270729

[65] Carlson, L. E. et al. Tailoring Mind-Body therapies to individual needs: patients’ program preference and psychological traits as moderators of the effects of Mindfulness-Based cancer recovery and Supportive-Expressive therapy in distressed breast cancer survivors. *JNCI Monogr.* **2014** (50), 308–314 (2014). 10.1093/jncimonographs/lgu034 25749597

[66] Asbjørnsen, R. A. et al. Persuasive system design principles and behavior change techniques to stimulate motivation and adherence in electronic health interventions to support weight loss maintenance: scoping review. *J. Med. Internet Res.* **21** (6), e14265 (2019). 31228174 10.2196/14265 PMC6611151

[67] Kip, H. et al. The CeHRes roadmap 2.0: update of a holistic framework for Development, Implementation, and evaluation of eHealth technologies. *J. Med. Internet Res.* **27**, e59601 (2025). 39805104 10.2196/59601 PMC11773290

[68] Berg M, Silander E, Bove M, Johansson L, Nyman J, Hammerlid E. Fatigue in Long-Term Head and Neck Cancer Survivors From Diagnosis Until Five Years After Treatment. *Laryngoscope*.**133**(9), 2211-2221 (2023). 36695154 10.1002/lary.30534

[69] Saevarsdottir, S. R. & Gudmundsdottir, S. L. Mobile apps and quality of life in patients with breast cancer and survivors: systematic literature review. *J. Med. Internet Res.* **25**, e42852 (2023). 37494111 10.2196/42852 PMC10416803

[70] Helsedirektoratet *Nasjonalt Handlingsprogram Med Retningslinjer for diagnostikk, Behandling Og oppfølging Av Pasienter Med Brystkreft [nettdokument]* (Oslo, 2019).

[71] Andrykowski, M. A. & Manne, S. L. Are psychological interventions effective and accepted by cancer patients? I. Standards and levels of evidence. *Ann. Behav. Med.* **32** (2), 93–97 (2006). 16972803 10.1207/s15324796abm3202_3

[72] Manne, S. L. & Andrykowski, M. A. Are psychological interventions effective and accepted by cancer patients? II. Using empirically supported therapy guidelines to decide. *Ann. Behav. Med.* **32** (2), 98–103 (2006). 16972804 10.1207/s15324796abm3202_4

[73] Cillessen, L. et al. Mindfulness-based interventions for psychological and physical health outcomes in cancer patients and survivors: A systematic review and meta-analysis of randomized controlled trials. *Psychooncology* **28** (12), 2257–2269 (2019). 31464026 10.1002/pon.5214 PMC6916350

[74] Buneviciene, I. et al. Can mHealth interventions improve quality of life of cancer patients? A systematic review and meta-analysis. *Crit. Rev. Oncol. Hematol.* **157**, 103123 (2021). 33190065 10.1016/j.critrevonc.2020.103123 PMC7574857

[75] Sandell, R. et al. Credibility clusters, preferences, and helpfulness beliefs for specific forms of psychotherapy. *Psychol. Psychotherapy: Theory Res. Pract.* **84** (4), 425–441 (2011). 10.1111/j.2044-8341.2010.02010.x 22903884

[76] Williams, R. et al. Patient preference in psychological treatment and associations with self-reported outcome: National cross-sectional survey in England and Wales. *BMC Psychiatry*. **16** (1), 4 (2016). 26768890 10.1186/s12888-015-0702-8 PMC4714467

[77] Koszycki, D. et al. Does treatment preference affect outcome in a randomized trial of a mindfulness intervention versus cognitive behaviour therapy for social anxiety disorder? *Clin. Psychol. Psychother.* **29** (2), 652–663 (2022). 34390076 10.1002/cpp.2658

[78] Ledel Solem, I. K. et al. A User-Centered approach to an Evidence-Based electronic health pain management intervention for people with chronic pain: design and development of EPIO. *J. Med. Internet Res.* **22** (1), e15889 (2020). 31961331 10.2196/15889 PMC7001051

[79] Milne-Ives, M. et al. The conceptualisation and measurement of engagement in digital health. *Internet Interv*. **36**, 100735 (2024). 38558760 10.1016/j.invent.2024.100735 PMC10979253

[80] Miller, W. R. & Rollnick, S. *Motivational interviewing: helping people change. Applications of motivational interviewing* 3rd edn. (The Guilford Press, 2013).

[81] Walsh, E. A. et al. If we build it, will they come? A scoping review of objective engagement metrics in asynchronous psychosocial telehealth interventions for breast cancer survivors. *Clin. Psychol. Rev.* **107**, 102374 (2024). 38171138 10.1016/j.cpr.2023.102374

[82] Rincon, E. et al. Mobile phone apps for quality of life and Well-Being assessment in breast and prostate cancer patients: systematic review. *JMIR Mhealth Uhealth*. **5** (12), e187 (2017). 29203459 10.2196/mhealth.8741 PMC5735250

