# Supplementary material for: Testing two digital stress-management interventions in a randomized controlled trial of breast cancer patients
Source: Sci Rep. 2025 Nov 6;15:38966. doi: 10.1038/s41598-025-22889-0 (PMC12592344; doi:10.1038/s41598-025-22889-0)
Supplement: Supplementary file 1 — Supplementary Material 1 [file 41598_2025_22889_MOESM1_ESM.pdf]

Testing two digital stress-management interventions in a randomized controlled trial of breast cancer patients

Karianne Svendsen [1,2], Lise Solberg Nes [3,4,5], Sigrid Leithe[1], Anders Meland[6], Ylva M. Gjelsvik[1], Elin Børøsund[3,7], Ine M. Larsson[1], Tor Åge Myklebust[1], Aina Balto[1], Christine M. Rygg[3], Cecilie E. Kiserud[8], Michael H. Antoni[9], Trudie Chalder[10], Ingvil Mjaaland [11], Linda E. Carlson [12], Hege R. Eriksen[13*] & Giske Ursin*[1,14,15]

[1] Cancer Registry of Norway, Norwegian Institute of Public Health, Oslo, Norway.

[2] Lipid Clinic, Oslo University Hospital, Oslo, Norway.

[3] Department of Digital Health Research, Division of Medicine, Oslo, University Hospital, Oslo, Norway.

[4] Institute of Clinical Medicine, Faculty of Medicine, University of Oslo, Oslo, Norway.

[5] Department of Psychiatry and Psychology, College of Medicine and Science, Rochester, USA.

[6]Department of Sport and Social Sciences, Norwegian School of Sport Sciences, Oslo, Norway.

[7] Department of Nursing and Health Sciences, Faculty of Health and Social Sciences, University of South-Eastern Norway, Drammen, Norway

[8] Department of Oncology, Oslo University hospital, Oslo, Norway.

[9] Department of Psychology, University of Miami, and Cancer Control Program, Sylvester Comprehensive Cancer Center, Miami, FL, US.

[10] Department of Psychological Medicine, King's College London, UK.

[11] Department of Oncology and Hematology, Stavanger University Hospital, Stavanger, Norway.

[12] Departments of Oncology and Psychology, University of Calgary, Canada.

[13] Department of Sport, Food and Natural Sciences, Western Norway University of Applied Sciences, Bergen, Norway.

[14] Department of Preventive Medicine, Keck School of Medicine, University of Southern California, Los Angeles, CA, USA.
[15] Department of Nutrition, University of Oslo, Oslo, Norway

*Contributed equally

Corresponding authors:

Karianne Svendsen and Giske Ursin
karianne.svendsen@fhi.no; giske.ursin@fhi.no

Supplementary file 1. Titles of modules (translated from Norwegian) in the StressProffen CBI and MBI apps.

Module titles of the two StressProffen interventions

CBI intervention

1. About stress
2. Stress, quality of life, and planning
3. Thoughts, emotions and self-care
4. Thoughts and visualization
5. Coping
6. Social support and humor
7. Anger management
8. Assertiveness and clear communication
9. Health behaviors and setting goals
10. Stress management and continued practice

MBI intervention

1. About stress
2. Stress and mindfulness
3. Stress, quality of life, and planning
4. Thoughts, emotions and self-care
5. Mindfulness and coping
6. Social support, humor and mindful movement
7. Anger management
8. Assertiveness and clear communication
9. Health behaviors and setting goals
10. Stress management and continued practice

Supplementary file 2. Examples of content in one intervention arm: the cognitive behavioral therapy intervention (CBI).

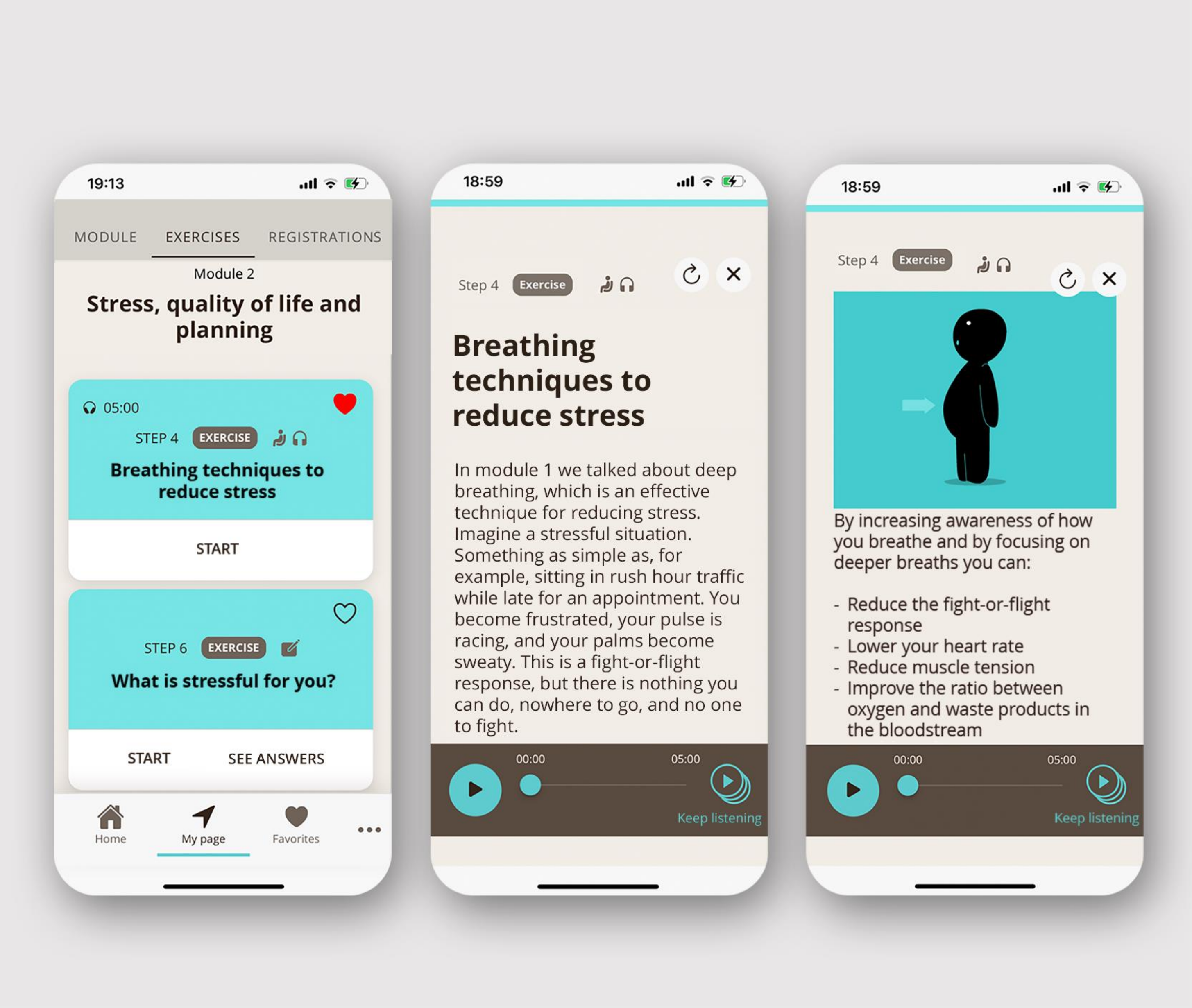

Supplementary file 3. Examples of content in one intervention arm: the mindfulness-based intervention (MBI).

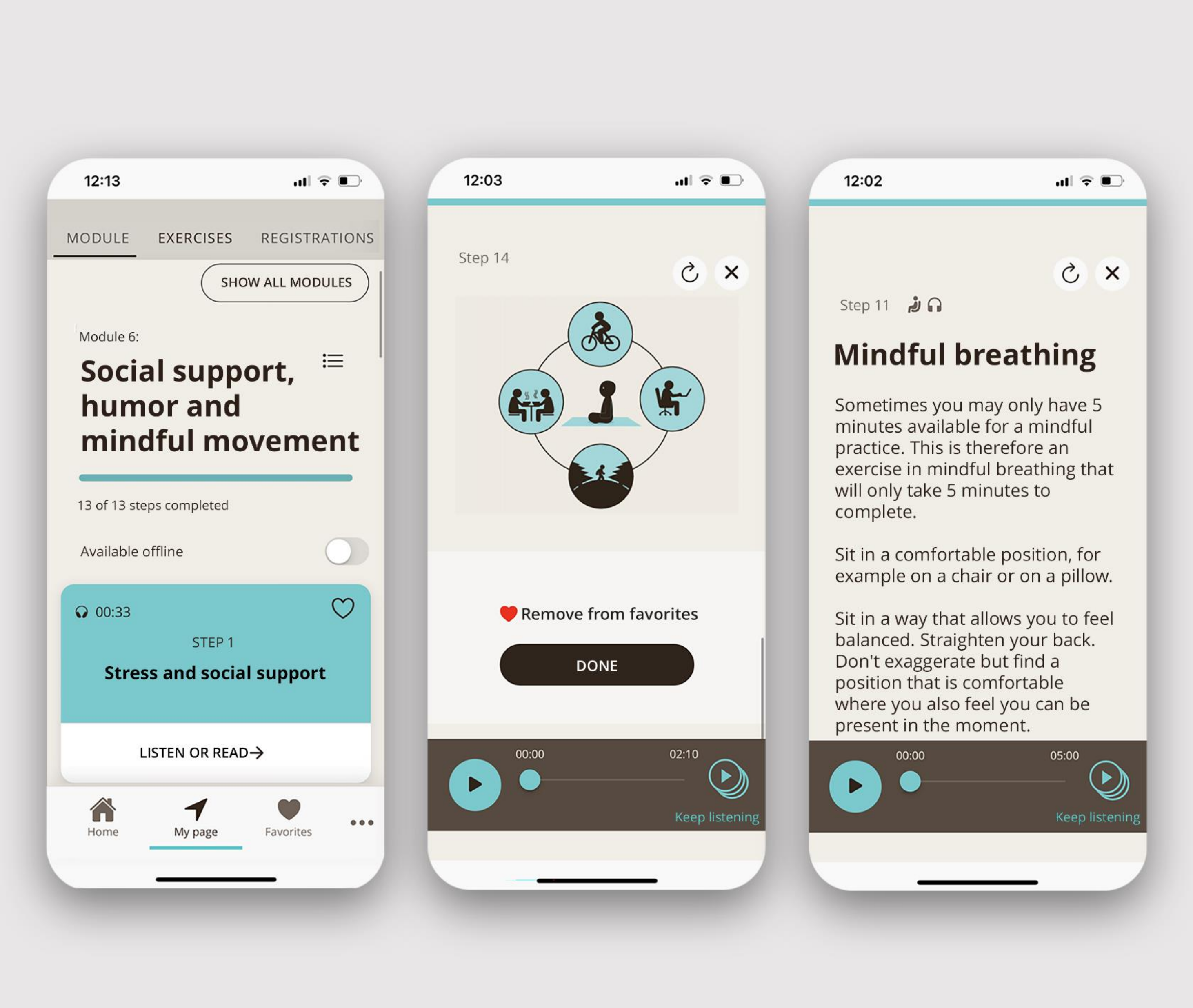

Supplementary file 4. Timeline for the Coping After Breast Cancer (CABC) trial. The Cancer registry of Norway patient reported outcome measures survey (CRN PROMs survey) is an ongoing survey sent to all breast cancer patients in Norway whereas the CABC survey is a CABC trial-specific survey covering the outcomes not assessed with the CRN PROMs survey.

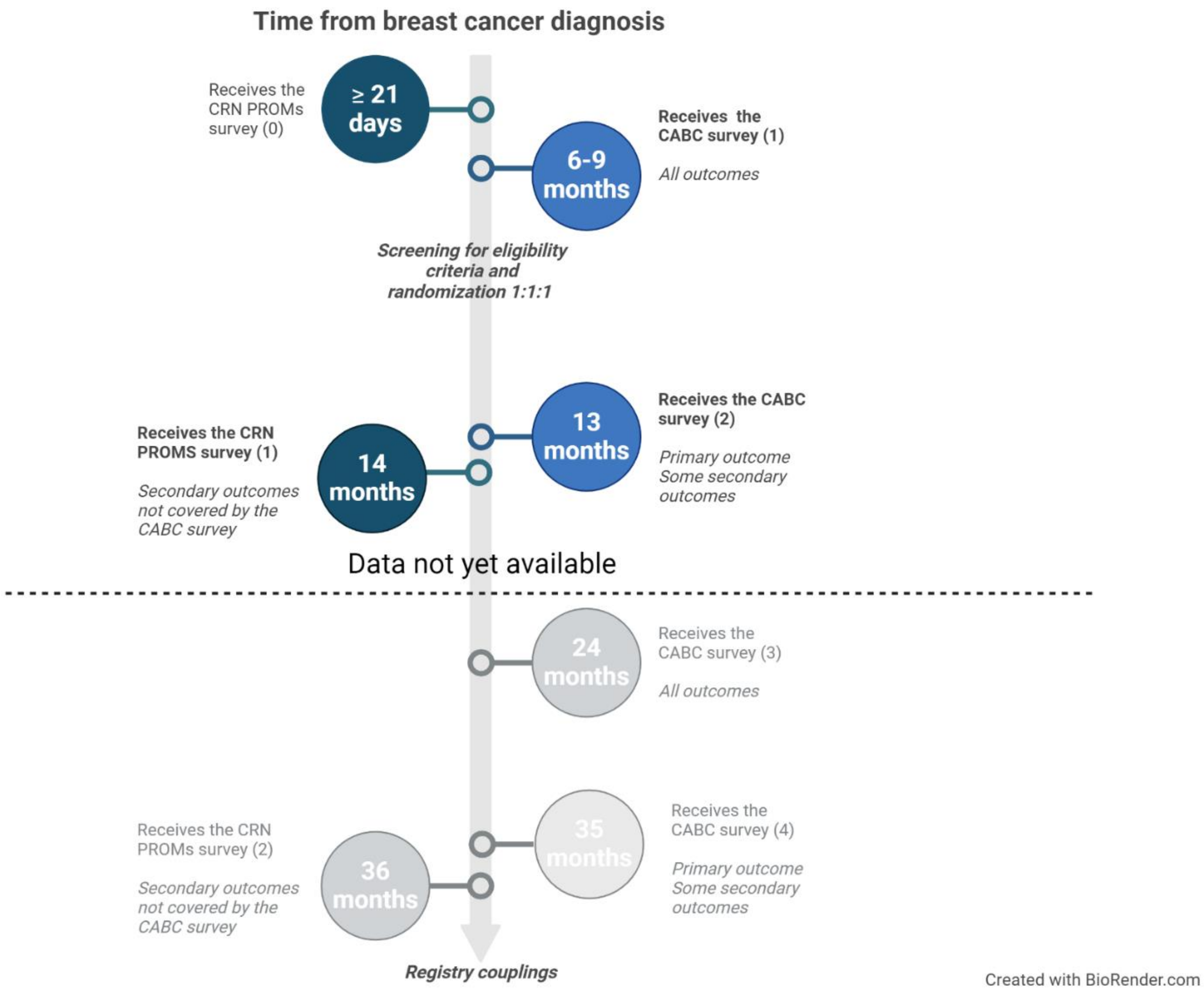

Supplementary file 5. Results from complete case analysis (attached as own file)

Supplementary file 6. Results from multi-adjusted model (attached as own file).
