# Supplementary material for: Testing two digital stress-management interventions in a randomized controlled trial of breast cancer patients
Source: Sci Rep. 2025 Nov 6;15:38966. doi: 10.1038/s41598-025-22889-0 (PMC12592344; doi:10.1038/s41598-025-22889-0)
Supplement: Supplementary file 2 — Supplementary Material 2 [file 41598_2025_22889_MOESM2_ESM.docx]

Testing two digital stress-management interventions in a randomized controlled trial of breast cancer patients

Karianne Svendsen ^1,2^, Lise Solberg Nes ^3,4,5^, Sigrid Leithe^1^, Anders Meland^6^, Ylva M. Gjelsvik^1^, Elin Børøsund^3,7^, Ine M. Larsson^1^, Tor Åge Myklebust^1^, Aina Balto^1^, Christine M. Rygg^3^, Cecilie E. Kiserud^8^, Michael H. Antoni^9^, Trudie Chalder^10^, Ingvil Mjaaland ^11^, Linda E. Carlson ^12^, Hege R. Eriksen^13*^ & Giske Ursin*^1,14,15^

^1^ Cancer Registry of Norway, Norwegian Institute of Public Health, Oslo, Norway.

^2^ Lipid Clinic, Oslo University Hospital, Oslo, Norway.

^3^ Department of Digital Health Research, Division of Medicine, Oslo, University Hospital, Oslo, Norway.

^4^ Institute of Clinical Medicine, Faculty of Medicine, University of Oslo, Oslo, Norway.

^5^ Department of Psychiatry and Psychology, College of Medicine and Science, Rochester, USA.

^6^Department of Sport and Social Sciences, Norwegian School of Sport Sciences, Oslo, Norway.

^7^ Department of Nursing and Health Sciences, Faculty of Health and Social Sciences, University of South-Eastern Norway, Drammen, Norway

^8^ Department of Oncology, Oslo University hospital, Oslo, Norway.

^9^ Department of Psychology, University of Miami, and Cancer Control Program, Sylvester Comprehensive Cancer Center, Miami, FL, US.

^10^ Department of Psychological Medicine, King's College London, UK.

^11^ Department of Oncology and Hematology, Stavanger University Hospital, Stavanger, Norway.

^12^ Departments of Oncology and Psychology, University of Calgary, Canada.

^13^ Department of Sport, Food and Natural Sciences, Western Norway University of Applied Sciences, Bergen, Norway.

^14^ Department of Preventive Medicine, Keck School of Medicine, University of Southern California, Los Angeles, CA, USA.
^15^ Department of Nutrition, University of Oslo, Oslo, Norway

*Contributed equally

Corresponding authors:

Karianne Svendsen and Giske Ursin
[karianne.svendsen@fhi.no](mailto:kasv@kreftregisteret.no); [giske.ursin@fhi.no](mailto:giske.ursin@fhi.no)

| **Supplementary file 5.** Results from complete case analysis of mean levels and mean difference in primary and secondary outcomes between the  CBI and MBI interventions and the control group. | | | | | | | | | | |
| --- | --- | --- | --- | --- | --- | --- | --- | --- | --- | --- |
|  | **CBI  (n = 99)** | | **MBI  (n = 105)** | | **Control group  (n = 109)** | | **CBI vs. control group difference** | | **MBI vs. control group difference** | |
|  | *Mean* | *95% CI* | Mean | 95% CI | Mean | 95% CI | MD | 95% CI | MD | 95% CI |
| **Perceived stress (PSS-10)** |  |  |  |  |  |  |  |  |  |  |
| Baseline | *15.23* | 13.76, 16.70 | 13.79 | 12.34, 15.23 | 14.26 | 12.85, 15.67 | 0.97 | -1.06, 3.00 | -0.47 | -2.49, 1.55 |
| 6 month follow-up | *14.42* | 12.97, 15.86 | 12.98 | 11.56, 14.40 | 13.50 | 12.11, 14.89 | 0.92 | -1.09, 2.92 | -0.52 | -2.51, 1.47 |
| Change adjusted for baseline | *-0.58* | -1.58, 0.43 | -0.99 | -1.98, 0.00 | -0.80 | -1.77, 0.16 | 0.23 | -1.17, 1.62 | -0.18 | -1.57, 1.20 |
| **HRQoL (RAND-36)** |  |  |  |  |  |  |  |  |  |  |
| **General health** |  |  |  |  |  |  |  |  |  |  |
| Baseline | *60.88* | *56.85, 64.90* | 64.65 | 60.68, 68.62 | 64.43 | 60.56, 68.30 | -3.55 | -9.14, 2.03 | 0.22 | -5.32, 5.76 |
| 6 month follow-up | *63.14* | *58.98, 67.30* | 67.20 | 63.10, 71.30 | 62.33 | 58.34, 66.33 | 0.81 | -4.96, 6.58 | 4.87 | -0.86, 10.59 |
| Change adjusted for baseline | *1.64* | *-1.25, 4.53* | 2.88 | 0.04, 5.72 | -1.82 | -4.60, 0.95 | 3.46 | -0.55, 7.47 | 4.70 | 0.73, 8.67 |
| **Physical functioning** |  |  |  |  |  |  |  |  |  |  |
| Baseline | *76.74* | *72.53, 80.96* | 79.72 | 75.53, 83.91 | 77.73 | 73.70, 81.77 | -0.99 | -6.83, 4.84 | 1.99 | -3.83, 7.81 |
| 6 month follow-up | *79.83* | *75.79, 83.88* | 81.61 | 77.59, 85.63 | 79.95 | 76.07, 83.82 | -0.12 | -5.72, 5.49 | 1.66 | -3.92, 7.25 |
| Change adjusted for baseline | *2.73* | *0.09, 5.37* | 2.34 | -0.29, 4.97 | 2.13 | -0.40, 4.65 | 0.60 | -3.05, 4.26 | 0.21 | -3.43, 3.86 |
| **Role-physical** |  |  |  |  |  |  |  |  |  |  |
| Baseline | *35.00* | *26.26, 43.74* | 39.75 | 31.23, 48.27 | 36.21 | 27.97, 44.45 | -1.21 | -13.23, 10.80 | 3.54 | -8.32, 15.39 |
| 6 month follow-up | *47.37* | *38.68, 56.06* | 51.50 | 43.03, 59.97 | 49.77 | 41.58, 57.95 | -2.40 | -14.33, 9.54 | 1.73 | -10.04, 13.51 |
| Change adjusted for baseline | *11.57* | *4.66, 18.49* | 12.84 | 6.10, 19.58 | 13.24 | 6.73, 19.75 | -1.66 | -11.16, 7.83 | -0.40 | -9.77, 8.97 |
| **Role-emotional** |  |  |  |  |  |  |  |  |  |  |
| Baseline | *61.86* | *53.52, 70.19* | 69.00 | 60.79, 77.21 | 68.22 | 60.29, 76.16 | -6.37 | -17.88, 5.14 | 0.78 | -10.64, 12.19 |
| 6 month follow-up | *65.29* | *57.22, 73.37* | 71.67 | 63.71, 79.62 | 73.83 | 66.14, 81.52 | -8.54 | -19.69, 2.61 | -2.17 | -13.23, 8.90 |
| Change adjusted for baseline | *0.67* | *-6.72, 8.06* | 4.20 | -3.07, 11.48 | 6.68 | -0.35, 13.71 | -6.01 | -16.22, 4.20 | -2.47 | -12.58, 7.63 |
| **Vitality** |  |  |  |  |  |  |  |  |  |  |
| Baseline | *44.58* | *39.94, 49.22* | 48.85 | 44.33, 53.37 | 46.84 | 42.45, 51.23 | -2.26 | -8.65, 4.12 | 2.01 | -4.29, 8.31 |
| 6 month follow-up | *50.37* | *45.76, 54.98* | 53.70 | 49.21, 58.19 | 48.87 | 44.50, 53.23 | 1.50 | -4.85, 7.85 | 4.83 | -1.43, 11.10 |
| Change adjusted for baseline | *5.14* | *1.89, 8.39* | 5.45 | 2.29, 8.62 | 2.04 | -1.03, 5.11 | 3.10 | -1.37, 7.57 | 3.41 | -1.00, 7.82 |
| **Mental health** |  |  |  |  |  |  |  |  |  |  |
| Baseline | *71.51* | *68.11, 74.90* | 75.80 | 72.45, 79.15 | 73.52 | 70.26, 76.79 | -2.02 | -6.73, 2.70 | 2.28 | -2.40, 6.95 |
| 6 month follow-up | *73.44* | *70.29, 76.60* | 77.28 | 74.17, 80.39 | 76.00 | 72.97, 79.03 | -2.56 | -6.93, 1.82 | 1.28 | -3.06, 5.62 |
| Change adjusted for baseline | *1.21* | *-1.02, 3.45* | 2.22 | 0.02, 4.42 | 2.44 | 0.30, 4.58 | -1.23 | -4.32, 1.87 | -0.22 | -3.29, 2.85 |
| **Social functioning** |  |  |  |  |  |  |  |  |  |  |
| Baseline | *63.14* | *58.18, 68.11* | 71.62 | 66.73, 76.52 | 65.83 | 61.06, 70.61 | -2.69 | -9.58, 4.20 | 5.79 | -1.05, 12.63 |
| 6 month follow-up | *70.62* | *65.92, 75.32* | 76.25 | 71.62, 80.88 | 72.50 | 67.98, 77.02 | -1.88 | -8.40, 4.64 | 3.75 | -2.72, 10.22 |
| Change adjusted for baseline | *6.08* | *2.53, 9.62* | 6.39 | 2.90, 9.89 | 6.27 | 2.88, 9.67 | -0.20 | -5.10, 4.71 | 0.12 | -4.76, 5.00 |
| **Bodily pain** |  |  |  |  |  |  |  |  |  |  |
| Baseline | *61.20* | *56.26, 66.14* | 66.05 | 61.21, 70.89 | 64.11 | 59.43, 68.79 | -2.91 | -9.72, 3.89 | 1.94 | -4.79, 8.67 |
| 6 month follow-up | *65.39* | *60.23, 70.55* | 67.05 | 62.00, 72.10 | 65.42 | 60.53, 70.31 | -0.03 | -7.14, 7.08 | 1.63 | -5.40, 8.66 |
| Change adjusted for baseline | *3.34* | *-0.60, 7.29* | 1.72 | -2.14, 5.58 | 1.40 | -2.33, 5.13 | 1.94 | -3.49, 7.37 | 0.32 | -5.05, 5.69 |
| **Mindfulness  (FFMQ-15)** |  |  |  |  |  |  |  |  |  |  |
| Baseline | *12.34* | *11.76, 12.91* | 13.10 | 12.53, 13.68 | 12.98 | 12.42, 13.53 | -0.64 | -1.44, 0.15 | 0.13 | -0.67, 0.92 |
| 6 month follow-up | *12.81* | *12.24, 13.38* | 13.67 | 13.10, 14.25 | 13.02 | 12.47, 13.57 | -0.20 | -0.99, 0.59 | 0.66 | -0.14, 1.45 |
| Change adjusted for baseline | *0.35* | *-0.03, 0.74* | 0.65 | 0.26, 1.04 | 0.08 | -0.29, 0.45 | 0.27 | -0.27, 0.81 | 0.56 | 0.03, 1.10 |
| **TOMCATS** |  |  |  |  |  |  |  |  |  |  |
| **Coping** |  |  |  |  |  |  |  |  |  |  |
| Baseline | *3.13* | *3.03, 3.24* | 3.22 | 3.12, 3.32 | 3.09 | 2.98, 3.19 | 0.05 | -0.10, 0.19 | 0.13 | -0.01, 0.28 |
| 6 month follow-up | *3.05* | *2.95, 3.16* | 3.21 | 3.11, 3.31 | 3.13 | 3.03, 3.23 | -0.08 | -0.23, 0.06 | 0.08 | -0.07, 0.22 |
| Change adjusted for baseline | *-0.09* | *-0.19, 0.01* | 0.04 | -0.06, 0.13 | 0.01 | -0.08, 0.10 | -0.10 | -0.23, 0.03 | 0.02 | -0.11, 0.16 |
| **Helplessness** |  |  |  |  |  |  |  |  |  |  |
| Baseline | *1.98* | *1.84, 2.12* | 1.83 | 1.69, 1.97 | 1.91 | 1.77, 2.04 | 0.07 | -0.13, 0.27 | -0.08 | -0.27, 0.12 |
| 6 month follow-up | *2.01* | *1.87, 2.15* | 1.85 | 1.71, 1.99 | 1.90 | 1.77, 2.03 | 0.11 | -0.08, 0.30 | -0.05 | -0.24, 0.14 |
| Change adjusted for baseline | *0.06* | *-0.05, 0.17* | -0.01 | -0.12, 0.10 | -0.01 | -0.11, 0.10 | 0.07 | -0.08, 0.22 | -0.00 | -0.15, 0.15 |
| **Hopelessness** |  |  |  |  |  |  |  |  |  |  |
| Baseline | *1.59* | *1.47, 1.71* | 1.48 | 1.36, 1.60 | 1.46 | 1.34, 1.58 | 0.13 | -0.04, 0.30 | 0.02 | -0.14, 0.19 |
| 6 month follow-up | *1.54* | *1.42, 1.66* | 1.46 | 1.34, 1.57 | 1.50 | 1.38, 1.61 | 0.04 | -0.12, 0.21 | -0.04 | -0.20, 0.12 |
| Change adjusted for baseline | *-0.01* | *-0.11, 0.09* | -0.04 | -0.13, 0.06 | 0.02 | -0.08, 0.11 | -0.03 | -0.16, 0.11 | -0.05 | -0.19, 0.08 |
| **Global Fatigue (CFQ-11)** |  |  |  |  |  |  |  |  |  |  |
| Baseline | 20.20 | 19.07 to 21.32 | 19.49 | 18.36 to 20.63 | 20.17 | 19.10 to 21.23 | 0.03 | -1.52 to 1.58 | -0.67 | -2.23 to 0.89 |
| 6 month follow-up | 18.48 | 17.31 to 19.66 | 17.49 | 16.30 to 18.69 | 18.51 | 17.39 to 19.63 | -0.02 | -1.65 to 1.60 | -1.01 | -2.65 to 0.62 |
| Change adjusted for baseline | -1.65 | -2.47 to -0.84 | -2.11 | -2.94 to -1.29 | -1.61 | -2.38 to -0.83 | -0.05 | -1.17 to 1.08 | -0.51 | -1.64 to 0.63 |
| **Anxiety and Depression (PHQ-4)** |  |  |  |  |  |  |  |  |  |  |
| Baseline | *3.08* | *2.56, 3.60* | 2.54 | 2.02, 3.05 | 2.99 | 2.49, 3.49 | 0.09 | -0.63, 0.81 | -0.46 | -1.17, 0.26 |
| 6 month follow-up | *2.92* | *2.44, 3.39* | 2.25 | 1.78, 2.72 | 2.65 | 2.19, 3.10 | 0.27 | -0.39, 0.93 | -0.40 | -1.05, 0.26 |
| Change adjusted for baseline | *-0.09* | *-0.43, 0.26* | -0.41 | -0.75, -0.07 | -0.30 | -0.63, 0.03 | 0.21 | -0.26, 0.69 | -0.11 | -0.59, 0.36 |
| Sleep (hours) |  |  |  |  |  |  |  |  |  |  |
| Baseline | *7.08* | *6.83, 7.33* | 7.16 | 6.91, 7.40 | 7.20 | 6.97, 7.44 | -0.12 | -0.47, 0.22 | -0.05 | -0.39, 0.30 |
| 6 month follow-up | *7.38* | *6.94, 7.82* | 7.24 | 6.80, 7.68 | 7.24 | 6.82, 7.66 | 0.14 | -0.47, 0.74 | -0.01 | -0.62, 0.60 |
| Change adjusted for baseline | *0.29* | *-0.10, 0.68* | 0.08 | -0.31, 0.47 | 0.05 | -0.32, 0.42 | 0.24 | -0.30, 0.78 | 0.03 | -0.50, 0.57 |
| **Sleep  (summary score)** |  |  |  |  |  |  |  |  |  |  |
| Baseline | *17.91* | *15.79, 20.02* | 15.78 | 13.67, 17.88 | 16.53 | 14.52, 18.54 | 1.37 | -1.54, 4.29 | -0.76 | -3.67, 2.15 |
| 6 month follow-up | *16.95* | *14.88, 19.01* | 15.02 | 12.97, 17.07 | 15.34 | 13.37, 17.30 | 1.61 | -1.24, 4.46 | -0.32 | -3.16, 2.53 |
| Change adjusted for baseline | *-0.60* | *-2.05, 0.84* | -1.04 | -2.48, 0.40 | -1.26 | -2.63, 0.12 | 0.65 | -1.35, 2.65 | 0.21 | -1.78, 2.20 |

*Complete case analysis*

M; mean

MD; mean difference

PSS; perceived stress scale.

HRQoL; Health- related quality of life

RAND-36; RAND corporation 36-item Short Form health Survey

FFMQ; Five Facet Mindfulness Questionnaire

TOMCATS; Theoretically Originated Measure of the Cognitive Activation Theory of Stress

CFQ-11: Chalder fatigues questionnaire 11 items

PHQ; Patient health questionnaire
