# Supplementary material for: Testing two digital stress-management interventions in a randomized controlled trial of breast cancer patients
Source: Sci Rep. 2025 Nov 6;15:38966. doi: 10.1038/s41598-025-22889-0 (PMC12592344; doi:10.1038/s41598-025-22889-0)
Supplement: Supplementary file 3 — Supplementary Material 3 [file 41598_2025_22889_MOESM3_ESM.docx]

# Testing two digital stress-management interventions in a randomized controlled trial of breast cancer patients

Karianne Svendsen ^1,2^, Lise Solberg Nes ^3,4,5^, Sigrid Leithe^1^, Anders Meland^6^, Ylva M. Gjelsvik^1^, Elin Børøsund^3,7^, Ine M. Larsson^1^, Tor Åge Myklebust^1^, Aina Balto^1^, Christine M. Rygg^3^, Cecilie E. Kiserud^8^, Michael H. Antoni^9^, Trudie Chalder^10^, Ingvil Mjaaland ^11^, Linda E. Carlson ^12^, Hege R. Eriksen^13*^ & Giske Ursin*^1,14,15^

^1^ Cancer Registry of Norway, Norwegian Institute of Public Health, Oslo, Norway.

^2^ Lipid Clinic, Oslo University Hospital, Oslo, Norway.

^3^ Department of Digital Health Research, Division of Medicine, Oslo, University Hospital, Oslo, Norway.

^4^ Institute of Clinical Medicine, Faculty of Medicine, University of Oslo, Oslo, Norway.

^5^ Department of Psychiatry and Psychology, College of Medicine and Science, Rochester, USA.

^6^Department of Sport and Social Sciences, Norwegian School of Sport Sciences, Oslo, Norway.

^7^ Department of Nursing and Health Sciences, Faculty of Health and Social Sciences, University of South-Eastern Norway, Drammen, Norway

^8^ Department of Oncology, Oslo University hospital, Oslo, Norway.

^9^ Department of Psychology, University of Miami, and Cancer Control Program, Sylvester Comprehensive Cancer Center, Miami, FL, US.

^10^ Department of Psychological Medicine, King's College London, UK.

^11^ Department of Oncology and Hematology, Stavanger University Hospital, Stavanger, Norway.

^12^ Departments of Oncology and Psychology, University of Calgary, Canada.

^13^ Department of Sport, Food and Natural Sciences, Western Norway University of Applied Sciences, Bergen, Norway.

^14^ Department of Preventive Medicine, Keck School of Medicine, University of Southern California, Los Angeles, CA, USA.
^15^ Department of Nutrition, University of Oslo, Oslo, Norway

*Contributed equally

Corresponding authors:

Karianne Svendsen and Giske Ursin
[karianne.svendsen@fhi.no](mailto:kasv@kreftregisteret.no); [giske.ursin@fhi.no](mailto:giske.ursin@fhi.no)

| **Supplementary file 6.** Results from the multi-adjusted model of mean levels and mean difference in primary and secondary outcomes between the  CBI and MBI interventions and the control group. | | | | | | | | | | |
| --- | --- | --- | --- | --- | --- | --- | --- | --- | --- | --- |
|  | **CBI (n = 140)** | | **MBI (n = 143)** | | **Control group (n = 147)** | | **CBI vs. control group difference** | | **MBI vs. control group difference** | |
|  | M | 95% CI | M | 95% CI | M | 95% CI | MD | 95% CI | MD | 95% CI |
| **Perceived stress (PSS-10)** |  |  |  |  |  |  |  |  |  |  |
| Baseline | 16.31 | 15.05 , 17.57 | 15.38 | 14.13 , 16.62 | 15.49 | 14.26 , 16.71 | 0.82 | -0.94 , 2.59 | -0.11 | -1.86 , 1.65 |
| 6 month follow-up | 14.97 | 13.56 , 16.38 | 14.33 | 12.96 , 15.69 | 14.74 | 13.42 , 16.07 | 0.23 | -1.71 , 2.16 | -0.41 | -2.32 , 1.49 |
| Change adjusted for baseline | -1.17 | -2.27 , -0.07 | -1.15 | -2.21 , -0.09 | -0.81 | -1.83 , 0.22 | -0.36 | -1.86 , 1.14 | -0.34 | -1.81 , 1.13 |
| **HRQoL (RAND-36)** |  |  |  |  |  |  |  |  |  |  |
| **General health** |  |  |  |  |  |  |  |  |  |  |
|  |  |  |  |  |  |  |  |  |  |  |
| Baseline | 59.86 | 56.44 , 63.29 | 61.87 | 58.50 , 65.24 | 61.66 | 58.36 , 64.95 | -1.79 | -6.57 , 2.98 | 0.21 | -4.51 , 4.94 |
| 6 month follow-up | 61.72 | 57.83 , 65.62 | 64.03 | 60.05 , 68.02 | 60.17 | 56.45 , 63.88 | 1.56 | -3.76 , 6.87 | 3.87 | -1.56 , 9.30 |
| Change adjusted for baseline | 1.52 | -1.48 , 4.52 | 2.36 | -0.84 , 5.56 | -1.36 | -4.22 , 1.50 | 2.88 | -1.15 , 6.91 | 3.71 | -0.49 , 7.91 |
| **Physical functioning** |  |  |  |  |  |  |  |  |  |  |
| Baseline | 75.65 | 72.42 , 78.88 | 78.23 | 75.04 , 81.42 | 77.33 | 74.24 , 80.42 | -1.68 | -6.18 , 2.83 | 0.90 | -3.56 , 5.37 |
| 6 month follow-up | 78.71 | 75.09 , 82.33 | 80.15 | 76.49 , 83.82 | 78.04 | 74.61 , 81.47 | 0.67 | -4.36 , 5.71 | 2.12 | -2.93 , 7.16 |
| Change adjusted for baseline | 2.74 | 0.03 , 5.46 | 2.18 | -0.60 , 4.96 | 0.76 | -1.77 , 3.29 | 1.98 | -1.77 , 5.72 | 1.42 | -2.33 , 5.16 |
| **Role-physical** |  |  |  |  |  |  |  |  |  |  |
| Baseline | 33.18 | 26.35 , 40.01 | 37.03 | 30.33 , 43.74 | 33.52 | 27.00 , 40.03 | -0.33 | -9.83 , 9.16 | 3.52 | -5.89 , 12.93 |
| 6 month follow-up | 45.98 | 38.18 , 53.79 | 46.46 | 38.26 , 54.66 | 47.20 | 39.51 , 54.88 | -1.21 | -12.18 , 9.75 | -0.74 | -12.26 , 10.79 |
| Change adjusted for baseline | 12.18 | 5.30 , 19.06 | 10.52 | 3.17 , 17.87 | 13.21 | 6.43 , 19.98 | -1.03 | -10.62 , 8.56 | -2.69 | -12.99 , 7.62 |
| **Role-emotional** |  |  |  |  |  |  |  |  |  |  |
| Baseline | 57.07 | 49.64 , 64.50 | 62.34 | 55.04 , 69.63 | 61.27 | 54.17 , 68.37 | -4.20 | -14.54 , 6.14 | 1.07 | -9.16 , 11.30 |
| 6 month follow-up | 63.85 | 55.41 , 72.29 | 67.11 | 58.86 , 75.36 | 71.39 | 63.72 , 79.07 | -7.54 | -19.00 , 3.91 | -4.28 | -15.61 , 7.04 |
| Change adjusted for baseline | 4.81 | -3.17 , 12.78 | 6.06 | -1.70 , 13.81 | 10.75 | 3.54 , 17.96 | -5.94 | -16.66 , 4.77 | -4.69 | -15.27 , 5.88 |
| **Vitality** |  |  |  |  |  |  |  |  |  |  |
| Baseline | 43.65 | 39.70 , 47.59 | 46.31 | 42.44 , 50.18 | 45.18 | 41.40 , 48.96 | -1.53 | -7.02 , 3.96 | 1.13 | -4.30 , 6.56 |
| 6 month follow-up | 49.32 | 44.88 , 53.75 | 50.94 | 46.57 , 55.30 | 47.10 | 43.00 , 51.20 | 2.22 | -3.81 , 8.24 | 3.84 | -2.26 , 9.93 |
| Change adjusted for baseline | 5.29 | 1.79 , 8.78 | 4.97 | 1.57 , 8.36 | 1.96 | -1.10 , 5.01 | 3.33 | -1.25 , 7.92 | 3.01 | -1.66 , 7.69 |
| **Mental health** |  |  |  |  |  |  |  |  |  |  |
| Baseline | 69.38 | 66.27 , 72.50 | 71.63 | 68.56 , 74.69 | 71.08 | 68.07 , 74.08 | -1.69 | -6.03 , 2.65 | 0.55 | -3.75 , 4.85 |
| 6 month follow-up | 72.21 | 69.11 , 75.31 | 74.17 | 71.08 , 77.25 | 74.09 | 71.14 , 77.05 | -1.88 | -6.12 , 2.35 | 0.07 | -4.18 , 4.32 |
| Change adjusted for baseline | 2.37 | 0.02 , 4.73 | 2.85 | 0.46 , 5.25 | 3.14 | 0.90 , 5.39 | -0.77 | -3.94 , 2.40 | -0.29 | -3.48 , 2.90 |
| **Social functioning** |  |  |  |  |  |  |  |  |  |  |
| Baseline | 62.13 | 57.78 , 66.47 | 66.90 | 62.62 , 71.18 | 64.61 | 60.42 , 68.80 | -2.49 | -8.55 , 3.58 | 2.29 | -3.72 , 8.30 |
| 6 month follow-up | 69.56 | 65.03 , 74.09 | 72.37 | 67.68 , 77.06 | 70.88 | 66.51 , 75.24 | -1.32 | -7.51 , 4.88 | 1.49 | -4.91 , 7.89 |
| Change adjusted for baseline | 6.55 | 2.90 , 10.20 | 6.31 | 2.39 , 10.24 | 6.28 | 2.78 , 9.78 | 0.27 | -4.62 , 5.16 | 0.03 | -5.13 , 5.19 |
| **Bodily pain** |  |  |  |  |  |  |  |  |  |  |
| Baseline | 60.49 | 56.45 , 64.54 | 63.44 | 59.47 , 67.40 | 62.41 | 58.54 , 66.28 | -1.92 | -7.55 , 3.72 | 1.03 | -4.54 , 6.60 |
| 6 month follow-up | 64.41 | 59.53 , 69.29 | 64.26 | 59.48 , 69.04 | 63.61 | 59.01 , 68.20 | 0.80 | -5.96 , 7.56 | 0.65 | -6.06 , 7.36 |
| Change adjusted for baseline | 3.35 | -0.78 , 7.48 | 1.28 | -2.76 , 5.31 | 1.30 | -2.51 , 5.11 | 2.05 | -3.58 , 7.69 | -0.02 | -5.64 , 5.59 |
| **Mindfulness (FFMQ-15)** |  |  |  |  |  |  |  |  |  |  |
| Baseline | 12.35 | 11.88 , 12.83 | 12.87 | 12.40 , 13.34 | 12.77 | 12.31 , 13.23 | -0.41 | -1.08 , 0.25 | 0.10 | -0.56 , 0.77 |
| 6 month follow-up | 12.75 | 12.23 , 13.28 | 13.40 | 12.89 , 13.90 | 12.87 | 12.37 , 13.38 | -0.12 | -0.86 , 0.61 | 0.52 | -0.19 , 1.24 |
| Change adjusted for baseline | 0.31 | -0.08 , 0.71 | 0.58 | 0.19 , 0.96 | 0.13 | -0.25 , 0.51 | 0.18 | -0.38 , 0.75 | 0.45 | -0.09 , 0.98 |
| **TOMCATS** |  |  |  |  |  |  |  |  |  |  |
| **Coping** |  |  |  |  |  |  |  |  |  |  |
| Baseline | 3.11 | 3.01 , 3.20 | 3.18 | 3.08 , 3.27 | 3.04 | 2.95 , 3.13 | 0.07 | -0.06 , 0.20 | 0.14 | 0.01 , 0.27 |
| 6 month follow-up | 3.01 | 2.91 , 3.11 | 3.18 | 3.07 , 3.28 | 3.09 | 2.99 , 3.19 | -0.08 | -0.23 , 0.06 | 0.08 | -0.06 , 0.23 |
| Change adjusted for baseline | -0.10 | -0.19 , 0.00 | 0.04 | -0.06 , 0.14 | 0.02 | -0.07 , 0.11 | -0.11 | -0.25 , 0.02 | 0.02 | -0.11 , 0.16 |
| **Helplessness** |  |  |  |  |  |  |  |  |  |  |
| Baseline | 2.05 | 1.93 , 2.17 | 1.96 | 1.85 , 2.08 | 2.00 | 1.88 , 2.11 | 0.05 | -0.12 , 0.22 | -0.03 | -0.20 , 0.13 |
| 6 month follow-up | 2.05 | 1.92 , 2.19 | 1.98 | 1.84 , 2.11 | 1.97 | 1.84 , 2.10 | 0.08 | -0.11 , 0.27 | 0.01 | -0.18 , 0.20 |
| Change adjusted for baseline | 0.02 | -0.09 , 0.14 | -0.00 | -0.12 , 0.12 | -0.03 | -0.14 , 0.08 | 0.05 | -0.11 , 0.21 | 0.03 | -0.13 , 0.19 |
| **Hopelessness** |  |  |  |  |  |  |  |  |  |  |
| Baseline | 1.64 | 1.53 , 1.76 | 1.57 | 1.46 , 1.68 | 1.57 | 1.47 , 1.68 | 0.07 | -0.08 , 0.22 | -0.01 | -0.16 , 0.15 |
| 6 month follow-up | 1.59 | 1.47 , 1.71 | 1.51 | 1.40 , 1.63 | 1.59 | 1.47 , 1.71 | -0.01 | -0.18 , 0.17 | -0.08 | -0.25 , 0.09 |
| Change adjusted for baseline | -0.04 | -0.14 , 0.06 | -0.07 | -0.17 , 0.03 | 0.01 | -0.10 , 0.11 | -0.05 | -0.19 , 0.10 | -0.08 | -0.21 , 0.06 |
| **Global Fatigue (CFQ-11)** |  |  |  |  |  |  |  |  |  |  |
| Baseline | 20.44 | 19.53 , 21.35 | 19.37 | 18.48 , 20.27 | 20.07 | 19.19 , 20.95 | 0.37 | -0.90 , 1.64 | -0.70 | -1.95 , 0.56 |
| 6 month follow-up | 18.68 | 17.66 , 19.71 | 17.37 | 16.28 , 18.46 | 18.63 | 17.65 , 19.62 | 0.05 | -1.38 , 1.49 | -1.26 | -2.74 , 0.22 |
| Change adjusted for baseline | -1.61 | -2.43 , -0.80 | -2.18 | -3.10 , -1.26 | -1.41 | -2.18 , -0.63 | -0.21 | -1.35 , 0.93 | -0.77 | -1.98 , 0.43 |
| **Anxiety and Depression (PHQ-4)** |  |  |  |  |  |  |  |  |  |  |
| Baseline | 3.39 | 2.91 , 3.86 | 3.04 | 2.57 , 3.51 | 3.18 | 2.72 , 3.64 | 0.20 | -0.46 , 0.87 | -0.14 | -0.80 , 0.52 |
| 6 month follow-up | 3.10 | 2.64 , 3.57 | 2.70 | 2.24 , 3.16 | 2.84 | 2.40 , 3.28 | 0.26 | -0.38 , 0.91 | -0.14 | -0.78 , 0.49 |
| Change adjusted for baseline | -0.21 | -0.57 , 0.14 | -0.40 | -0.76 , -0.05 | -0.35 | -0.69 , -0.01 | 0.14 | -0.37 , 0.64 | -0.05 | -0.54 , 0.43 |
| **Sleep (hours)** |  |  |  |  |  |  |  |  |  |  |
| Baseline | 7.02 | 6.79 , 7.24 | 7.10 | 6.88 , 7.33 | 7.18 | 6.96 , 7.39 | -0.16 | -0.48 , 0.16 | -0.07 | -0.39 , 0.24 |
| 6 month follow-up | 7.28 | 6.75 , 7.81 | 7.22 | 6.82 , 7.63 | 7.25 | 6.86 , 7.63 | 0.03 | -0.62 , 0.69 | -0.02 | -0.59 , 0.54 |
| Change adjusted for baseline | 0.25 | -0.25 , 0.75 | 0.12 | -0.25 , 0.49 | 0.09 | -0.26 , 0.44 | 0.16 | -0.44 , 0.77 | 0.03 | -0.48 , 0.54 |
| Sleep (summary score) |  |  |  |  |  |  |  |  |  |  |
| **Baseline** | 19.02 | 17.13 , 20.90 | 17.75 | 15.89 , 19.60 | 17.66 | 15.84 , 19.47 | 1.36 | -1.27 , 3.99 | 0.09 | -2.52 , 2.69 |
| 6 month follow-up | 17.59 | 15.53 , 19.66 | 16.77 | 14.77 , 18.76 | 16.18 | 14.17 , 18.18 | 1.42 | -1.47 , 4.31 | 0.59 | -2.27 , 3.45 |
| Change adjusted for baseline | -1.17 | -2.75 , 0.41 | -1.09 | -2.59 , 0.42 | -1.62 | -3.15 , -0.08 | 0.44 | -1.77 , 2.65 | 0.53 | -1.65 , 2.70 |
| Missing data handled by multiple imputation. All values were adjusted for e a-priori defined potential confounders age groups (18-44, 45-54, 55+), educational level (low and high), having children <18 years (yes/no), BMI groups (under- and normal weight, overweight or obese), breast cancer stadium (DCIS + stage 1, stage 2 and 3 or missing) and alcohol use.  M; mean  MD; mean difference | | | | | | | | | | |

PSS; perceived stress scale.

HRQoL; Health- related quality of life

RAND-36; RAND corporation 36-item Short Form health Survey

FFMQ; Five Facet Mindfulness Questionnaire

TOMCATS; Theoretically Originated Measure of the Cognitive Activation Theory of Stress

CFQ-11: Chalder fatigues questionnaire 11 items

PHQ; Patient health questionnaire
